# Feasibility, image quality, and analytic usability of mobile 64 mT ultra-low-field MRI for infant brain imaging at 3 and 12 months in southern Malawi

**DOI:** 10.1016/j.jmro.2026.100225

**Published:** 2026-09

**Authors:** Maclean Vokhiwa, Karen Chetcuti, Frederik Lange, Niall J. Bourke, Louise Randall, Steven Greenstein, Marc Seal, Richard Beare, Adam Hussain, Padma Rao, Emil Ljungberg, Francesco Padormo, John Rogers, Pip Torelli, Steve Williams, Derek K. Jones, Sean C.L. Deoni, Sant-Rayn Pasricha, Kamija S. Phiri, Eric Umar, Unity Consortium

**Affiliations:** a Training & Research Unit of Excellence (TRUE), Blantyre, Malawi; b Kamuzu University of Health Sciences (KUHeS), Blantyre, Malawi; c Department of Radiology, Kamuzu University of Health Sciences (KUHeS), Blantyre, Malawi; d Centre for Integrative Neuroimaging (OxCIN), FMRIB, Nuffield Department of Clinical Neurosciences, University of Oxford, United Kingdom; e Centre for Neuroimaging Sciences, Department of Psychology and Neuroscience, King’s College London, London, United Kingdom; f Population Health and Immunity, Walter and Eliza Hall Institute of Medical Research, Parkville, Victoria, Australia; g Developmental Imaging, Murdoch Children’s Research Institute, The Royal Children’s Hospital, Victoria, Australia; h Department of Medical Imaging, The Royal Children’s Hospital, Parkville, Victoria, Australia; i Department of Neuroimaging, King’s College London, London, United Kingdom; j Department of Medical Radiation Physics, Lund University, Lund, Sweden; k Hyperfine, Inc., Guilford, CT, USA; l Cardiff University Brain Research Imaging Centre (CUBRIC), Cardiff University, Cardiff, United Kingdom; m Maternal, Newborn, Child Nutrition & Health, Gates Foundation, Seattle, WA, United States; n Ultra-Low-Field Neuroimaging in The Young (UNITY), United Kingdom

**Keywords:** Ultra-low-field MRI, Infancy, Neuroimaging, Volumetry, Image quality, Feasibility, Africa

## Abstract

Mobile ultra-low-field MRI (ULF-MRI) could reduce inequities in infant neuroimaging in sub-Saharan Africa, but evidence of its feasibility, image quality, and analytic usability remains limited. We evaluated the feasibility, image quality, and analytic usability of mobile 64 mT ULF-MRI for non-sedated infant brain imaging at three and twelve months of age within a longitudinal trial platform in southern Malawi. Feasibility was assessed through visit-level scan uptake and sequence completion; image quality through artifact-based quality control (QC) and radiologist interpretability review; and analytic usability through multi-structure volumetry and correspondence across independent processing workflows.

Across 810 eligible visits (3 months: 410; 12 months: 400), 654 MRI sessions were completed (80.7%; 86.6% at 3 months; 74.8% at 12 months), with high sequence acquisition success among completed scans. Of 654 completed sessions, 495 entered artifact-based QC and 442/495 (89.3%) met predefined full-brain quality criteria for volumetric processing; motion-related degradation was the principal determinant of exclusion. Radiologist review (n = 426) rated 396/426 scans (93.0%) as analysable. Volumetry was derived for 215 infants at 3 months and 227 at 12 months, with 99 paired observations demonstrating measurable age-related differences in tissue volumes (grey matter +39.0 ± 7.2%; white matter +48.8± 9.5%). An independent cloud-based pipeline showed high correspondence for supratentorial tissue and total intracranial volume at 12 months (r ≈ 0.97).

These findings demonstrate that non-sedated infant ULF-MRI can acheive high uptake, interpretable imaging, and scalable volumetric processing in a low-resource setting. The results support its analytic readiness for integration into developmental neuroscience research where conventional MRI is unavailable.

## Highlights

• Mobile 64 mT MRI supported feasible non-sedated infant brain imaging in Malawi.
• High scan uptake and sequence completion at 3 and 12 months.
• Parallel QC pathways supported analytic and clinical image usability.
• Ultra-low-field MRI demonstrated analytic readiness for infant volumetry.
• End-to-end framework supports scalable infant neuroimaging in low-resource settings.

## Graphical abstract

**Figure:**
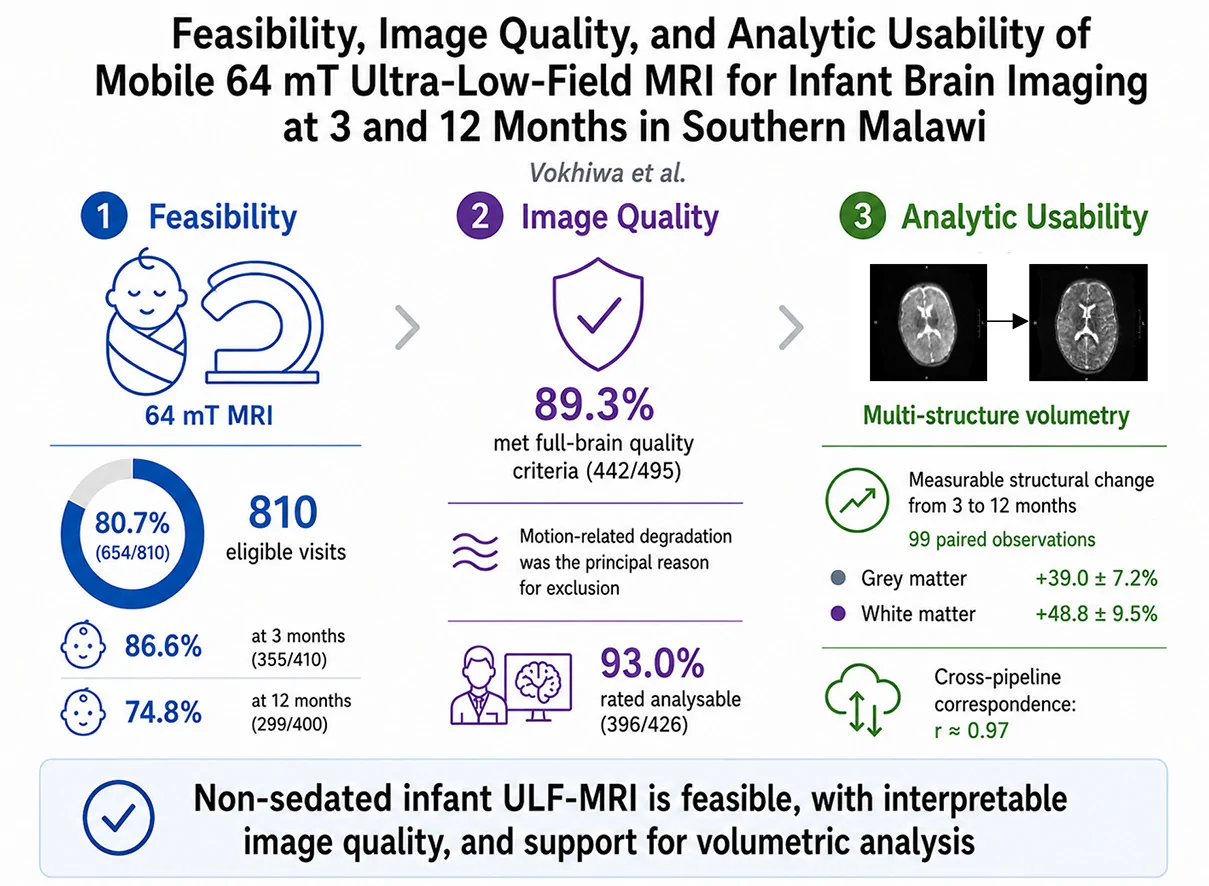

## 1 Introduction

MRI provides a powerful, non-invasive approach for examining early brain structure by enabling direct quantification of grey and white matter volumes, cortical morphology, and related structural biomarkers that are not captured by behavioural assessments alone [1]. These brain-based measures can detect early biological effects of nutritional, environmental, and clinical exposures before functional deficits become clinically apparent [2]. However, conventional high-field MRI systems are often unavailable or impractical in many sub-Saharan African (SSA) settings because of infrastructural requirements, high costs, space constraints, inconsistent electricity supply, and limited availability of trained personnel [3]. As a result, pediatric neuroimaging data remain scarce in regions where early-life nutritional and infectious risks are most prevalent.

Low- and ultra-low-field MRI systems (<100 mT) have emerged as a promising pathway to expand access to neuroimaging in low-resource settings. Mobile MRI systems, designed to operate within existing clinical infrastructure and under variable power conditions, reduce many of the logistical barriers associated with conventional scanners [4]. International collaborations, such as the Ultra-low-field Neuroimaging in The Young (UNITY) initiative, aim to advance safe, feasible, and standardized paediatric neuroimaging through harmonized operating procedures, quality assurance frameworks, and analytic pipelines tailored to low-field data [5]. Early feasibility studies have demonstrated the potential of mobile and ultra-low-field MRI for clinical and research applications, but evidence remains limited for infant brain imaging in SSA contexts [[6], [7], [8]]. Notably, infant neuroimaging presents distinct challenges compared with adult low-field MRI, including motion susceptibility, reliance on natural sleep, and constraints on scan duration, which remain underrepresented in existing low-field feasibility studies.

In this context, iron deficiency and anemia during pregnancy and early childhood represent a compelling example of conditions for which improved brain-based developmental measurement is needed. Iron deficiency and anemia remain major public health challenges globally, with a particularly high burden in SSA [9,10]. Prevalence estimates across the region indicate that a substantial proportion of infants and young children experience anemia in early life, while more than one-third of pregnant women are affected in many settings [11]. Maternal and early childhood iron deficiency anemia (IDA) have been consistently associated with adverse neurodevelopmental outcomes, including poorer cognitive, motor, and language performance, with effects that may persist into later childhood [[12], [13], [14], [15], [16]]. These associations are biologically plausible, as iron plays a critical role in neurodevelopmental processes such as myelination, neurotransmitter synthesis, and cerebral energy metabolism [17,18]. While the present study does not directly evaluate these clinical associations, this context highlights the importance of reliable, brain-based measures to better understand how early-life exposures may influence neurodevelopment. Despite this well-established risk profile, the magnitude, timing, and mechanistic pathways linking iron deficiency exposures to child neurodevelopment in SSA remain incompletely understood, in part due to limitations in how neurodevelopment is measured.

Within the sub-Saharan African literature, a key limitation of studies examining anemia and early child neurodevelopment concerns the measurement of developmental outcomes. Most studies rely on behavioural or psychometric assessments – typically developed in high-income settings, that are variably adapted for local cultural and linguistic contexts, with substantial heterogeneity in age of administration, translation, scoring, and reporting practices [19,20]. Reliability reporting is often sparse or absent, particularly following tool adaptation, introducing uncertainty in measurement precision and weakening confidence in both positive and null findings [21]. A recent systematic review of neurodevelopmental measurement among iron-deficient children in sub-Saharan Africa similarly identified major measurement heterogeneity, limited reporting of measurement reliability, and the near absence of neuroimaging-based assessments [22]. Critically, neuroimaging is almost entirely absent from this body of work, despite the biological relevance of brain structure and maturation as potential intermediates between early nutritional exposure and later developmental function [23]. Structural barriers including cost, infrastructure requirements, unreliable electricity, and limited access to trained personnel have constrained the integration of brain-based measures such as MRI [3,24,25]. Few studies explicitly evaluate image reliability, artifact burden, or analytic robustness, limiting confidence in the usability of low-field MRI brain-based measures even when acquisition is feasible. Together, these limitations restrict mechanistic inference, reduce comparability across studies and populations, and perpetuate inequities in how early brain development is measured and understood in African contexts.

In direct response to these measurement and access gaps, this study aimed to evaluate the research usability and image reliability of a mobile 64 mT ultra-low-field MRI system for infant brain imaging in Southern Malawi, embedded within a longitudinal pediatric neurodevelopmental study at 3 and 12 months of age. The primary objectives were to quantify scan attendance and sequence completion rates, assess full-brain image quality across required imaging planes, characterize the frequency and types of imaging artifacts, and evaluate whether ultra-low-field MRI datasets acquired in this setting support downstream volumetric processing suitable for developmental analyses in subsequent studies, including assessment of interpretability through radiologist review and correspondence of volumetric estimates across independent processing pipelines.

The study was guided by the following evaluative expectations:

1. Feasibility:
  Non-sedated infant MRI using a mobile 64 mT ultra-low-field system is operationally feasible in a low-resource SSA setting, achieving high scan uptake and sequence completion rates at 3 and 12 months of age.
2. Image quality:
  A substantial proportion of scans would meet predefined full-brain quality criteria suitable for downstream quantitative analysis.
3. Analytic usability:
  Ultra-low-field MRI datasets meeting quality criteria would support volumetric analysis across major brain compartments, yielding measurable age-related differences and consistent estimates across independent processing pipelines, suitable for use in subsequent developmental analyses.

By establishing practical protocols and testing these expectations in a low-resource SSA setting, this study sought to advance equitable, brain-based measurement approaches that complement behavioural assessments and strengthen the developmental evidence base in early childhood research, particularly by enabling the integration of neuroimaging-derived measures into studies of early-life exposures in under-resourced settings.

## 2 Materials and methods

### 2.1 Ethical approval

This study received ethical approval from the College of Medicine Research and Ethics Committee (COMREC; Protocol #P.11/22/3879). The parent randomized controlled trial within which this study was embedded was approved by the National Health Sciences Research Commission of Malawi (NHSRC; Protocol #20/11/2622). Written informed consent for MRI participation was obtained from all caregivers prior to neuroimaging. All procedures were conducted in accordance with the ethical principles outlined in the Declaration of Helsinki.

### 2.2 Study design and participants

This study reports the measurement and feasibility evaluation of a neuroimaging component nested within a larger randomized controlled trial evaluating preventive interventions for maternal iron deficiency anemia in Southern Malawi [26]. In the parent trial, neuroimaging was incorporated as an ancillary research component and did not influence trial allocation, intervention delivery, or trial outcomes. Parents or caregivers of infants were invited to participate in MRI scanning at scheduled trial follow-up visits conducted at 3 and 12 months of age. The study was conducted at a central referral hospital in Zomba District, a semi-urban region in southern Malawi.

Across the study period, 810 eligible infant visits were scheduled for neuroimaging at the 3- and 12-month timepoints (3 months: n = 410; 12 months: n = 400). MRI scanning was attempted at each eligible visit, resulting in 654 completed MRI sessions across both timepoints (3 months: n = 355; 12 months: n = 299), as defined by successful acquisition of at least one imaging sequence. This definition was used for feasibility analyses and did not determine eligibility for volumetric processing or radiologist review, which applied separate image-quality criteria as described in Section 2.9.

A subset of infants contributed MRI data at both visits, enabling paired within-subject comparisons across timepoints, while others contributed data at a single timepoint only. All infants included in this analysis were enrolled trial participants whose caregivers provided explicit consent for MRI scanning.

In addition to quantitative scan metrics, operational feasibility was monitored prospectively through structured weekly implementation review meetings, which documented workflow challenges, infrastructural constraints, data management issues, and participant-related considerations arising during routine study operations.

### 2.3 Imaging system and infrastructure

A mobile 64 mT ultra-low-field MRI (ULF-MRI) system (Hyperfine Swoop Mk1.7, Hyperfine, Inc., Guilford, CT, USA) was used for this study. The Swoop system has previously been implemented in Malawi [27], and a comparable system was deployed for the present study. The scanner incorporates a biplanar, whole-body unshielded gradient system and a solenoidal, linearly polarized volume radiofrequency (RF) coil with single-channel transmit and eight-channel receive architecture. The detachable head RF coil functions as a transmit/receive head coil with an eight-channel receive array. Gradient performance specifications include maximum gradient amplitudes of 24.9 mT/m (X), 24.4 mT/m (Y), and 25.7 mT/m (Z), with corresponding slew rates of 23, 22, and 67 T/m/s, respectively. The scanner is a compact, wheel-mounted unit weighing approximately 640 kg, with dimensions of 59 inches in height and 33 inches in width. It operates using standard electrical power. During the study period, backup power measures, including integration of an uninterruptible power supply (UPS) and backup generator support, were easily implemented and strengthened in response to observed power interruptions to improve continuity of operation. The scanner was installed in a dedicated imaging room within the Training and Research Unit of Excellence (TRUE) at Zomba Central Hospital, allowing for basic environmental control and minimization of external disturbances during infant scanning sessions. This room was not a conventional RF-shielded MRI suite; rather, it was a standard room designated for imaging activities, and no purpose-built RF shielding room or specialized MRI infrastructure was constructed for the study.

The system supports multiple imaging sequences, including T1-weighted and T2-weighted imaging, fluid-attenuated inversion recovery (FLAIR), and diffusion-weighted imaging (DWI). Scan operation was managed via a tablet-based interface, enabling sequence selection, scan initiation, real-time image monitoring, and data export. Following each scan session, de-identified DICOM image data were uploaded to the secure Hyperfine cloud platform for centralized data storage, image visualization, remote technical support, and access for subsequent image processing. Image datasets were also uploaded to the Flywheel research platform [28] for cloud-based processing and analysis.

Infrastructure performance, including power availability and data connectivity, was monitored throughout the study period. Operational issues were documented during weekly implementation review meetings and addressed through iterative deployment of backup systems and workflow adjustments.

### 2.4 Imaging protocol

Each scan session comprised five sequences: a localizer, three T2-weighted fast spin echo sequences (axial, coronal, and sagittal), and an axial T1-weighted sequence, with a total image acquisition time of approximately 11 minutes. The overall MRI session typically lasted approximately 25 minutes, including infant positioning, preparation, sequence setup, and image acquisition. The protocol was designed to provide complete structural brain coverage across orthogonal imaging planes while maintaining feasibility for non-sedated infant imaging. Sequence selection and acquisition duration were therefore balanced against anticipated infant motion, reliance on natural sleep, and limited tolerance for prolonged scanning. The T1-weighted sequence was acquired last because T2-weighted imaging provided the primary structural information required for image quality assessment and volumetric analyses in this age group, allowing priority to be given to the most analytically informative sequences should scanning be interrupted. Detailed acquisition parameters for all sequences are provided in Supplementary Table S1. Data were acquired using the system operating across multiple manufacturer software release versions during the study period (v8.2.2, v8.3.2, v8.4.1, v8.5.1, v8.6.0, and v8.6.1). Software version was recorded for each scan session to document acquisition conditions and support reproducibility. No known differences in image quality or downstream processing relevant to the present analyses were associated with the software release versions used during the study.

Consistent with established non-sedated pediatric neuroimaging protocols [2], no sedation, contrast agents, or invasive procedures were used. Infants were scanned while asleep or calm, either naturally or following feeding. Parents or caregivers remained present in the scanning room throughout image acquisition to support infant comfort and minimize movement during scanning.

### 2.5 Training and operational procedures

The on-site neuroimaging team consisted of two radiology technicians, two nurses, two early child development assessors, and one research scientist. A consulting radiologist and international collaborators provided remote technical and analytical support through the UNITY initiative and affiliated neuroimaging research groups.

The local team completed a three-day, hands-on training program in pediatric neuroimaging, with emphasis on non-sedated scanning, infant handling, and MRI safety. Training was supplemented by self-directed learning materials and ongoing remote mentorship.

To support consistent implementation across the study period, standardized operating procedures were established and followed throughout data collection. Weekly team meetings were conducted to review scanning challenges, optimize workflow, and monitor scan uptake and completion. These were complemented by structured operational review meetings involving the imaging team and study leadership, which documented implementation challenges and mitigation strategies related to participant flow, staffing, equipment use, and scan environment.

### 2.6 Operational monitoring and implementation documentation

Operational feasibility was assessed using a mixed-methods approach combining quantitative scan metrics with structured documentation of implementation processes. Weekly operational review meetings generated contemporaneous records of logistical challenges, infrastructural constraints, data pipeline performance, and participant-related factors. These records were categorized thematically and summarized to support interpretation of feasibility outcomes.

### 2.7 Participant preparation and scan environment

All neuroimaging sessions were preceded by confirmation of eligibility and re-affirmation of caregiver consent. Scanning was scheduled around infants’ natural sleep cycles to maximize stillness during image acquisition. Measures to reduce motion included swaddling, noise reduction, and dimmed lighting. The Hyperfine scanner produced a maximum average acoustic noise level of approximately 93 dB (Aeq) and a peak unweighted acoustic noise level of 116 dB during image acquisition.

Standard MRI safety procedures were followed throughout. The area within the scanner’s Gauss guard was maintained free of ferromagnetic objects, and non-essential equipment was positioned outside the immediate imaging zone. Although non-ferromagnetic items (e.g., mobile phones) are unlikely to affect image quality at ultra-low-field strengths, the scan environment was simplified to reduce potential sources of interference based on operational experience.

Infant safety and comfort were prioritized at all times. Caregivers remained present during scanning, and image acquisition was paused or discontinued if infants showed signs of distress or excessive movement. Observations related to infant settling, caregiver engagement, and scan environment were reviewed during operational meetings, with procedural adjustments implemented where recurrent challenges were identified.

### 2.8 Quality assurance and artifact control

A quality assurance framework was implemented using an MRI phantom optimized for the ultra-low-field system (Model 137, CaliberMRI, Boulder, CO, USA), scanned weekly according to a standardized UNITY protocol. These phantom scans were used to monitor scanner performance over time under variable operational conditions (e.g., ambient temperature, electromagnetic environment, and power source), rather than to adjust or calibrate system parameters [29]. Quality assurance scans were performed across different power configurations, including mains electricity, uninterruptible power supply (UPS), and generator power. These assessments were used to confirm stability of signal characteristics and sequence performance across operating conditions and to support interpretation of image quality outcomes, rather than as a primary analytic outcome of the present study.

Artifact mitigation was implemented through standardized operator procedures. These included consistent infant positioning, verification of appropriate head placement within the receive coil, and use of pre-scan checklists. Particular attention was given to (i) sagittal-plane streaking or streaming artifacts, (ii) intensity inhomogeneity observed in coronal acquisitions, and (iii) participant motion. Because these artifacts arise from a combination of participant movement, positioning, and local operating conditions, mitigation focused on operator-controlled steps, including ensuring appropriate head positioning within the coil, minimizing nearby sources of potential electromagnetic interference where feasible, and repeating affected sequences when image quality was compromised.

Artifact identification and classification were performed by trained reviewers using visual inspection. Scan sessions were flagged for review when quality criteria were not met, and artifacts were categorized by type and imaging plane.

### 2.9 Image quality assessment and processing

To address analytic usability, two complementary but distinct quality-control approaches were applied. A manual artifact-based assessment was used to determine suitability for volumetric analysis, while an independent radiologist-led assessment was used to determine clinical interpretability. These approaches were implemented independently in parallel to reflect different definitions of data usability within the study.

Following acquisition, image series underwent quality assessment using structured visual review procedures. Full-brain image quality was defined as clear visualization of the entire brain volume across the three orthogonally acquired T2-weighted images (axial, sagittal, and coronal). The axial T1-weighted sequence provided complementary anatomical information as part of the structural imaging examination. Image series that did not meet this criterion – due to incomplete coverage or severe artifacts – were excluded from volumetric analysis.

In parallel with the artifact-based quality assessment, a subset of completed MRI sessions underwent independent radiologist review to assess overall image interpretability and structural usability. Radiologists independently reviewed the full available image series using a separate structured review form and without access to the artifact-based quality assessment results. Scans were classified as analysable or not analysable based on the ability to visualize major intracranial structures sufficiently for structural assessment. For scans deemed not analysable, the primary reason was recorded using predefined categories (incomplete sequences or limited diagnostic series, motion-degraded or non-diagnostic studies, or other/unclear causes). Presence of motion artifact was recorded independently of analysability status. For analysable scans, structural findings across routinely assessed domains and recommendations for clinical follow-up, where applicable, were documented. Radiologist-derived classifications were used for interpretability and descriptive structural assessment only and did not determine inclusion in quantitative volumetric analyses. Radiologists did not review the resulting segmentation outputs. Segmentation was performed only on scans meeting full-brain quality criteria, and appropriateness of the derived volumetric measures was supported through standardized processing workflows together with evaluation of biological plausibility and correspondence across an independent processing pipeline.

Scans meeting full-brain quality criteria (i.e., complete visualization of the brain across all required imaging planes) were prepared using a standardized workflow optimized for infant ultra-low-field MRI. Processing comprised phantom-based retrospective distortion correction [30], super-resolution reconstruction using magnetic resonance reconstruction (MRR) [31] combined with modified Tikhonov deconvolution [32], followed by tissue segmentation using SynthSeg+ retrained on infant and neonatal MRI datasets [33]. Given the lower resolution and contrast of the 64 mT system, these methods were selected to account for reduced tissue contrast and increased image noise compared to conventional high-field MRI. Volumetric outputs included grey matter, white matter, cerebellar, ventricular, supratentorial, and total intracranial volumes, which were aggregated for subsequent descriptive and correspondence analyses.

In parallel, a cloud-based volumetric analysis pipeline was implemented using the Flywheel research data management platform. Raw DICOM images were ingested into Flywheel and automatically converted to NIfTI format for downstream research analysis. Image processing was performed using custom Flywheel “gears” built within Docker containers to ensure reproducibility, version control, and scalability across studies within the UNITY network. These containers integrate a combination of in-house and open-source neuroimaging tools and are publicly available for transparency and reuse (https://github.com/UNITY-Physics). Automated processing was triggered upon data ingestion and followed a standardized workflow. Axial T2-weighted images were first pre-processed using GAMBAS (Generalised-Hilbert Mamba Super-resolution) a hybrid convolutional neural network – state-space model developed for paediatric ultra-low-field MRI super-resolution [34]. The model was trained on paediatric ultra-low-field MRI datasets acquired in South Africa and Uganda across the developmental period from 3 months to 3 years of age, and the published pre-trained model weights were used in this study without additional retraining. GAMBAS was applied to axial T2-weighted images because the axial acquisition was performed first in the imaging protocol, was typically of the highest image quality, and was unaffected by the sagittal acquisition artefact present in earlier software versions. Images were then resampled to 1 × 1 × 1 mm isotropic resolution and processed using recon-all-clinical, a cortical surface reconstruction and volumetric segmentation pipeline designed for heterogeneous clinical MRI datasets [35]. Volumetric derivatives generated by this pipeline included cerebellar volume, supratentorial tissue volume, total intracranial volume, and additional cortical, subcortical, and cerebrospinal fluid compartments. Derivatives were aggregated across participants into a structured dataset for subsequent descriptive and correspondence analyses. Outputs from the Flywheel-based pipeline were evaluated for correspondence with volumetric estimates derived from the separate processing workflow.

Quality assessment and processing evaluations focused on consistency of full-brain quality classification and suitability for volumetric analysis.

### 2.10 Data management and statistical analysis

All imaging and participant data were stored in a secure, access-restricted digital environment. Data were de-identified prior to analysis. Summary statistics were generated for participant characteristics, scan attendance, sequence acquisition success, and image quality outcomes. Scan uptake was calculated as the proportion of eligible infants who underwent MRI at each scheduled visit.

Image quality yield was summarized as the proportion of scan sessions meeting full-brain quality criteria at each timepoint. Artifact profiles were described using frequencies and proportions by artifact type and imaging plane.

Descriptive statistics, including means, standard deviations, medians, interquartile ranges, and 95% confidence intervals where appropriate, were used to summarize participant characteristics and volumetric outcomes.

Operational implementation data from weekly review meetings were summarized descriptively and categorized thematically to contextualize quantitative feasibility outcomes. These data were not subjected to inferential analysis but were used to document implementation processes and mitigation strategies relevant to real-world deployment. Operational data pipeline disruptions and resolutions were prospectively documented and summarized.

To assess analytic usability across processing workflows, paired volumetric estimates derived from the separate processing workflow and the Flywheel-based pipeline were compared using Pearson correlation coefficients and two-way mixed-effects intraclass correlation coefficients (ICC[A,1]), with confidence intervals calculated to characterize both relative association and absolute agreement across processing environments.

## 3 Results

**Fig. 1:**
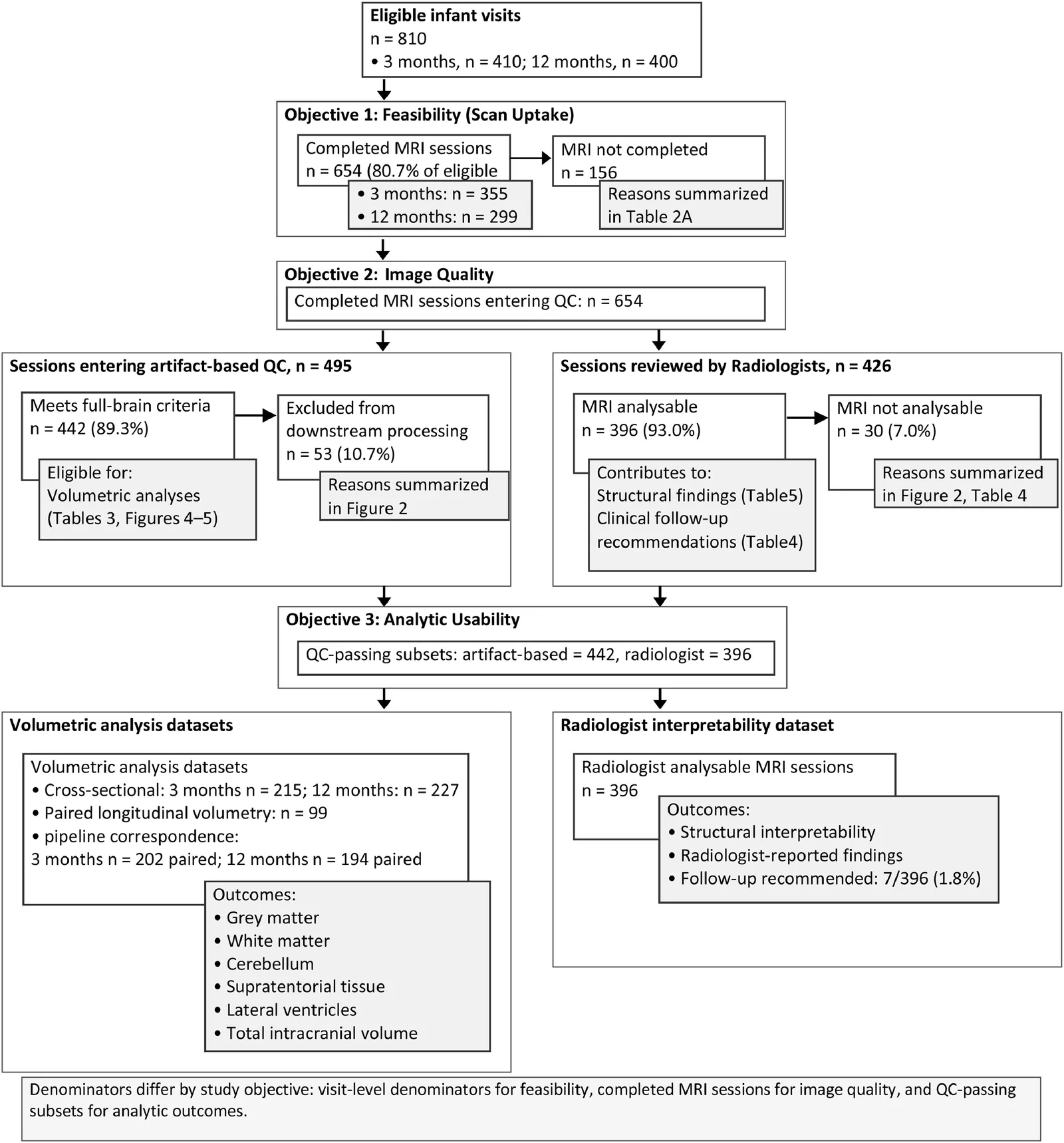
Participant flow and data inclusion across study objectives.

**Fig. 2:**
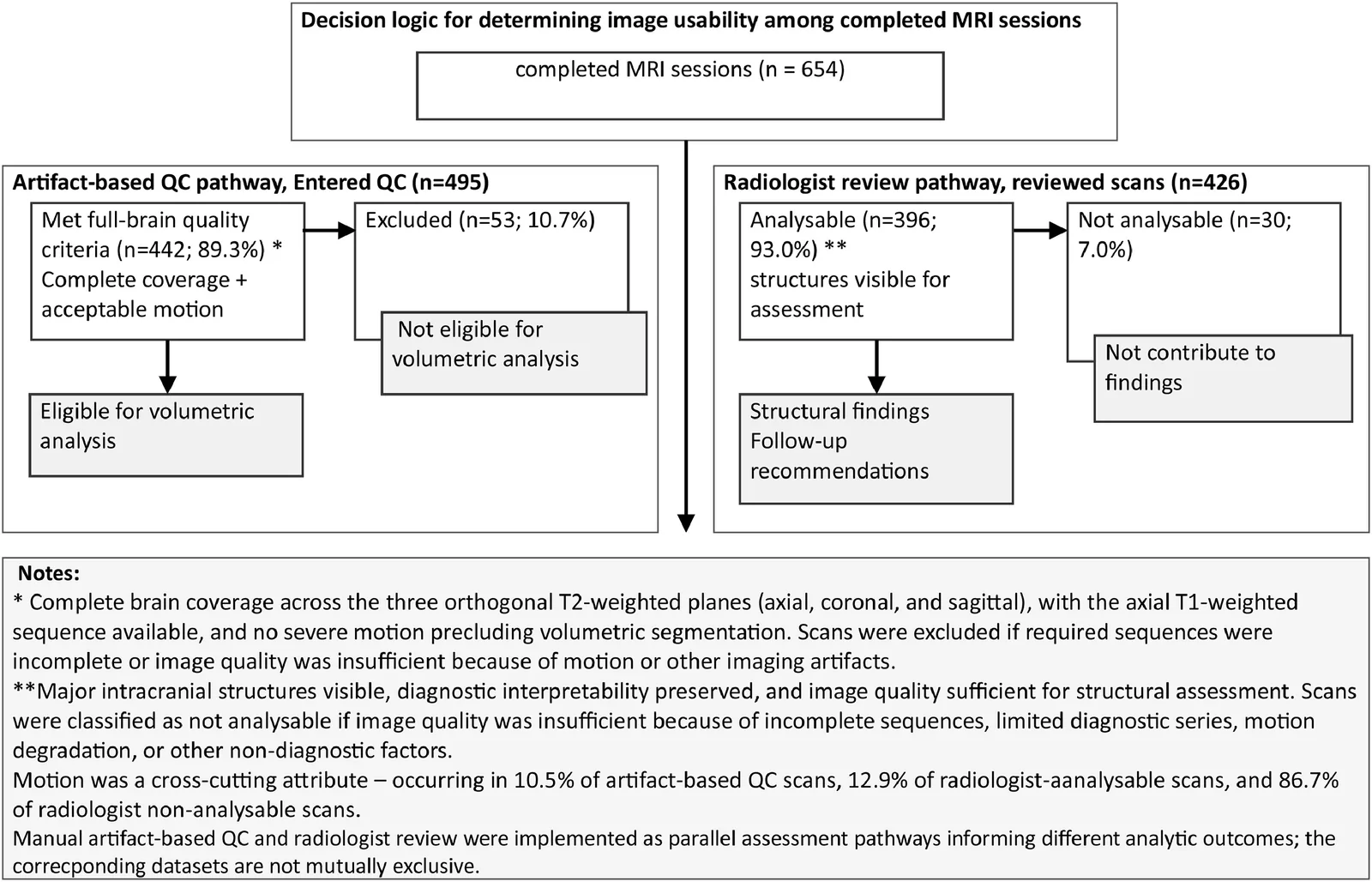
Image quality yield and parallel quality-control (QC) decision pathways for analytic usability in completed infant ultra-low-field MRI sessions.

Fig. 1 summarizes participant flow and data inclusion across study objectives, illustrating how visit-level eligibility, completed MRI sessions, and quality-controlled analytic subsets contribute to feasibility, image quality, and analytic usability outcomes. Fig. 2 outlines the parallel quality-control (QC) pathways applied to completed MRI sessions, comprising artifact-based and radiologist-based assessments used to determine data usability for downstream analyses.

Flow diagram summarizing eligible visits, completed MRI sessions, and downstream data inclusion across the three study objectives: feasibility (scan uptake), image quality assessment, and analytic usability. Visit-level denominators are used for feasibility analyses, completed MRI sessions form the denominator for image quality assessment, and quality-controlled subsets contribute to analytic outputs, including quantitative volumetry and radiologist-reported findings. The figure illustrates how differing denominators arise across objectives and analyses.

Schematic representation of parallel artifact-based and radiologist-based quality-control pathways applied independently to completed MRI sessions (n = 654). Manual artifact-based QC evaluated artifact burden and full-brain sequence completeness to determine suitability for volumetric analysis, while radiologist review assessed structural interpretability to classify scans as analysable or not analysable. These complementary, non-hierarchical pathways reflect distinct definitions of data usability for downstream analytic objectives.

### 3.1 Scan uptake, attendance, and sequence completion

Using visit-level denominators as shown in Fig. 1, scan uptake and sequence completion were assessed among eligible infant visits at the 3- and 12-month timepoints. Across the study period captured in the dataset, there were 810 eligible infant visits at the 3- and 12-month timepoints, of which 654 MRI sessions were completed (80.7%) (Table 1). Uptake was higher at 3 months (355/410; 86.6%) than at 12 months (299/400; 74.8%). Across weeks with scanning activity, throughput was stable, with a median of 7 scans per active week (IQR 4–9; range 1–19).

**Table 1:** Scan uptake and sequence completion by visit. Scan uptake and sequence-specific acquisition success for infant ultra-low-field MRI at 3 and 12 months of age. Scan uptake is reported as the proportion of eligible participant visits with a completed MRI session at each timepoint. Sequence completion rates represent the percentage of completed MRI sessions in which each imaging sequence was successfully acquired. Percentages for sequence completion are calculated among completed MRI sessions. Data are based on participant-visit records from the study dataset.

Timepoint (visit)	Eligible visits, n	MRI completed, n (%)	Localizer %	T2 axial %	T2 sagittal %	T2 coronal %	T1 axial %
3 months	410	355 (86.6)	94.6	99.4	95.8	96.6	91.0
12 months	400	299 (74.8)	95.0	99.7	96.7	97.0	95.0
**Overall**	**810**	**654 (80.7)**	–	–	–	–	–

– indicates not applicable

Among visits without a completed MRI session (n = 156), the most common reason for non-completion was infant restlessness or inability to settle, accounting for 81.8% of non-completions at 3 months and 78.2% at 12 months (Table 2A). Less frequent reasons included machine-related issues, power outages, caregiver choice, and other reasons (Table 2A).

**Table 2:** A, B. Reasons for MRI non-completion and characteristics of completed and non-completed visits. Primary reasons for MRI non-completion and descriptive characteristics of infant visits at 3 and 12 months of age. Panel A summarizes the primary reasons for non-completion among eligible visits without a completed MRI session. Panel B presents a descriptive comparison of sex and age at visit between visits with completed and non-completed MRI sessions, based on available data. Percentages are calculated using visit-specific denominators. No inferential statistical testing was performed.

Panel A						
Timepoint (visit)	MRI not completed, n	Infant restlessness / did not settle, n (%)	Machine problem, n (%)	Power outage, n (%)	Caregiver choice, n (%)	Other, n (%)
3 months	55	45 (81.8)	2 (3.6)	4 (7.3)	1 (1.8)	3 (5.5)
12 months	101	79 (78.2)	11 (10.9)	0 (0.0)	1 (1.0)	10 (9.9)
**Overall**	**156**	**124 (79.5)**	**13 (8.3)**	**4 (2.6)**	**2 (1.3)**	**13 (8.3)**

Panel B						
Timepoint	Group	Total n	Female, n (%) *	Median age, days (Q1-Q3) ⁎⁎		
**3 months**	MRI completed	355	169 (49.9%; n=339)	91 (89–94)		
	MRI not completed	55	24 (47.1%; n=51)	92 (89–94)		
**12 months**	MRI completed	299	132 (50.0%; n=264)	365 (362–368)		
	MRI not completed	101	50 (56.8%; n=88)	364 (360–366)		

⁎ Percentages were calculated among participants with available sex information. Values shown as “n=” indicate the denominator used for each percentage at the corresponding timepoint.

⁎⁎ Calculated among participants with available age data.

Sequence acquisition success rates were high among completed sessions. At 3 months, completion ranged from 91.0% for axial T1-weighted imaging to 99.4% for axial T2-weighted imaging; at 12 months, completion ranged from 95.0% for localizer and axial T1-weighted imaging to 99.7% for axial T2-weighted imaging (Table 1).

To explore potential bias due to missed scans, basic characteristics were compared descriptively between completed and non-completed visits where data were available (Table 2B). At both 3 and 12 months, sex distribution and age at visit were broadly similar between completed and non-completed visits. Detailed operational characteristics of MRI sessions including descriptive summaries of participant characteristics, infant behavior during scanning, and sequence-level acquisition details by visit are provided in Supplementary Table S2A-C.

Weekly operational review meetings documented practical implementation challenges and adaptive responses throughout the imaging period (Supplementary Table S3). Commonly reported issues related to participant flow and scheduling, including higher-than-anticipated attendance on specific clinic days, late arrivals from distant catchment areas, and extended waiting times, which at times affected infant settling prior to scanning. In addition, a temporary interruption to scanning occurred following a mechanical fault involving the power supply unit of the scanner’s integrated computer module. Inspection identified accumulation of dust and environmental debris, likely related to local environmental conditions and airflow from adjacent windows (Supplementary Fig. S1). Mitigation measures included cleaning of affected components, improved environmental sealing, and installation of a replacement power supply unit shipped by the manufacturer. Repairs were completed on site by local biomedical engineering personnel with remote technical support from Hyperfine staff, after which scanner performance was fully restored and imaging resumed without recurrence.

Environmental and infrastructural factors, including variable power availability, temporary reliance on alternative power sources, and room temperature management, required iterative mitigation through installation of uninterruptible power supply (UPS) support and temporary cooling measures. Data management challenges, such as intermittent image transfer and synchronization issues, were also noted and resolved through workflow adjustments and coordination with technical support. Early data pipeline disruptions related to image transfer and metadata synchronization were identified and resolved through workflow adaptations, supporting end-to-end feasibility (Supplementary Table S4). Caregiver engagement and acceptability were recurrent discussion points, leading to reinforcement of consent messaging, workflow streamlining, and the introduction of comfort measures to support infant settling and retention. Collectively, these observations indicate that the feasibility of infant ultra-low-field MRI in this setting depended not only on technical performance, but also on adaptive operational management within a real-world clinical research environment.

### 3.2 Image quality yield and artifact profile

**Fig. 3:**
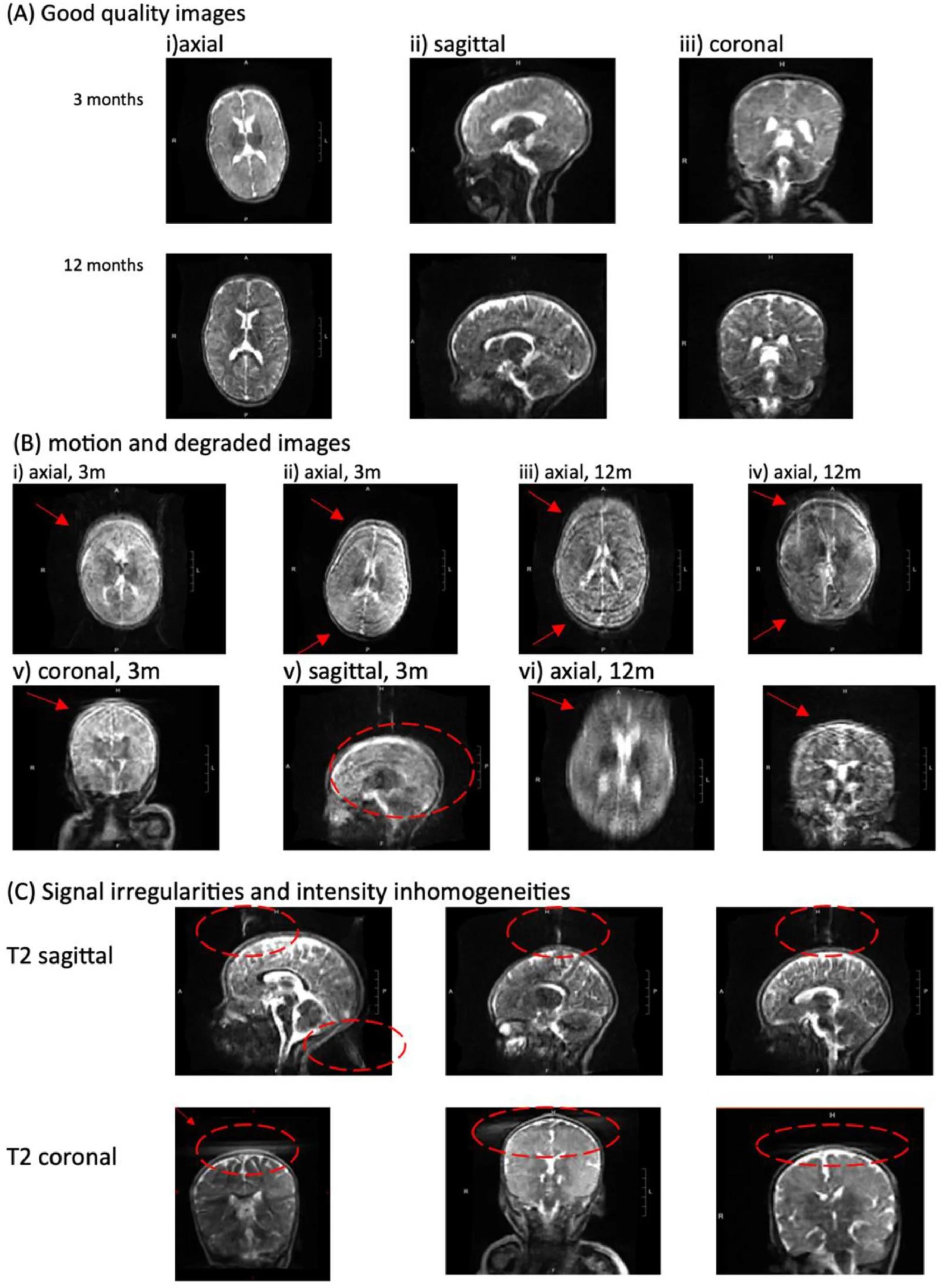
Representative image quality and artifact examples in infant ultra-low-field MRI.

Consistent with the study design, artifact-based QC and radiologist review were implemented as parallel evaluation pathways addressing different definitions of data usability (Fig. 2). Consequently, not all completed MRI sessions entered both pathways. Of the 654 completed MRI sessions, 495 (75.7%) entered the artifact-based quality-control workflow, within which 442/495 (89.3%) met full-brain quality criteria as described in Fig. 2, while 53/495 (10.7%) did not meet criteria for downstream volumetric processing due to insufficient image quality. The differing denominators for artifact-based QC and radiologist review therefore reflect the evaluation workflow rather than exclusions related to time constraints. Artifact profiling in the artifact-based QC pathway indicated that motion artifacts were present in 10.5% of sessions, while linear signal irregularities in the sagittal plane and coronal plane-specific intensity inhomogeneity artifacts were observed in 37.3% and 4.9% of sessions, respectively (Fig. 3). The underlying causes of the observed sagittal linear signal irregularities and coronal intensity inhomogeneity were not investigated in the present study. Accordingly, these artifacts were classified descriptively by type and imaging plane, and no conclusions can be drawn regarding their underlying mechanisms or whether they are specifically related to the ultra-low-field imaging environment.

Representative examples illustrating image quality and common artifacts observed in infant ultra-low-field MRI acquisitions. (A) Full-brain T2-weighted images demonstrating visualization of intracranial structures across axial, sagittal, and coronal planes. (B) Example of motion artefact associated with blurring and signal degradation across intracranial regions, resulting in exclusion from volumetric processing. (C) Example of linear signal irregularities in the sagittal plane and coronal plane-specific intensity inhomogeneity, which predominantly affect extracranial signal regions and typically preserve intracranial structure visibility for volumetric analysis.

Radiologist review was completed for 426 sessions. Most radiologist-reviewed scans were deemed interpretable, with 396/426 (93.0%) rated as MRI analysable, and 30/426 (7.0%) rated not analysable. Among non-analysable scans, the most common reasons were incomplete sequences or limited diagnostic series (17/30; 56.7%) and motion-degraded or non-diagnostic studies (i.e., image quality insufficient for structural assessment) (11/30; 36.7%), with a small proportion classified as other/unclear (2/30; 6.7%). Radiologist-identified motion artifact was present in 77/426 (18.1%) of reviewed sessions, occurring in 51/396 (12.9%) of analysable scans and 26/30 (86.7%) of non-analysable scans.

Collectively, these findings indicate that while artifacts were common, a substantial proportion of completed sessions met full-brain quality criteria on the artifact-based QC assessment and were interpretable on radiologist review, supporting the reliability and usability of infant ultra-low-field MRI acquisition in this setting.

### 3.3 Analytic usability of volumetric MRI data

#### 3.3.1 Volumetric analytic usability and age-related differences

Analytic usability of ultra-low-field MRI for volumetric outcomes was evaluated among QC-passing datasets identified through the artifact-based QC pathway shown in Fig. 2, with contributing denominators summarized in Fig. 1. Among MRI sessions meeting full-brain quality criteria, volumetric measures were successfully derived for multiple brain tissue compartments, demonstrating that acquired datasets can be processed into volumetric derivatives suitable for use in subsequent developmental analyses. Cross-sectional volumetric estimates were available for 215 infants at 3 months and 227 infants at 12 months, with a paired longitudinal subset of 99 infants contributing data at both timepoints (Tables 3A, 3B).

**Fig. 4:**
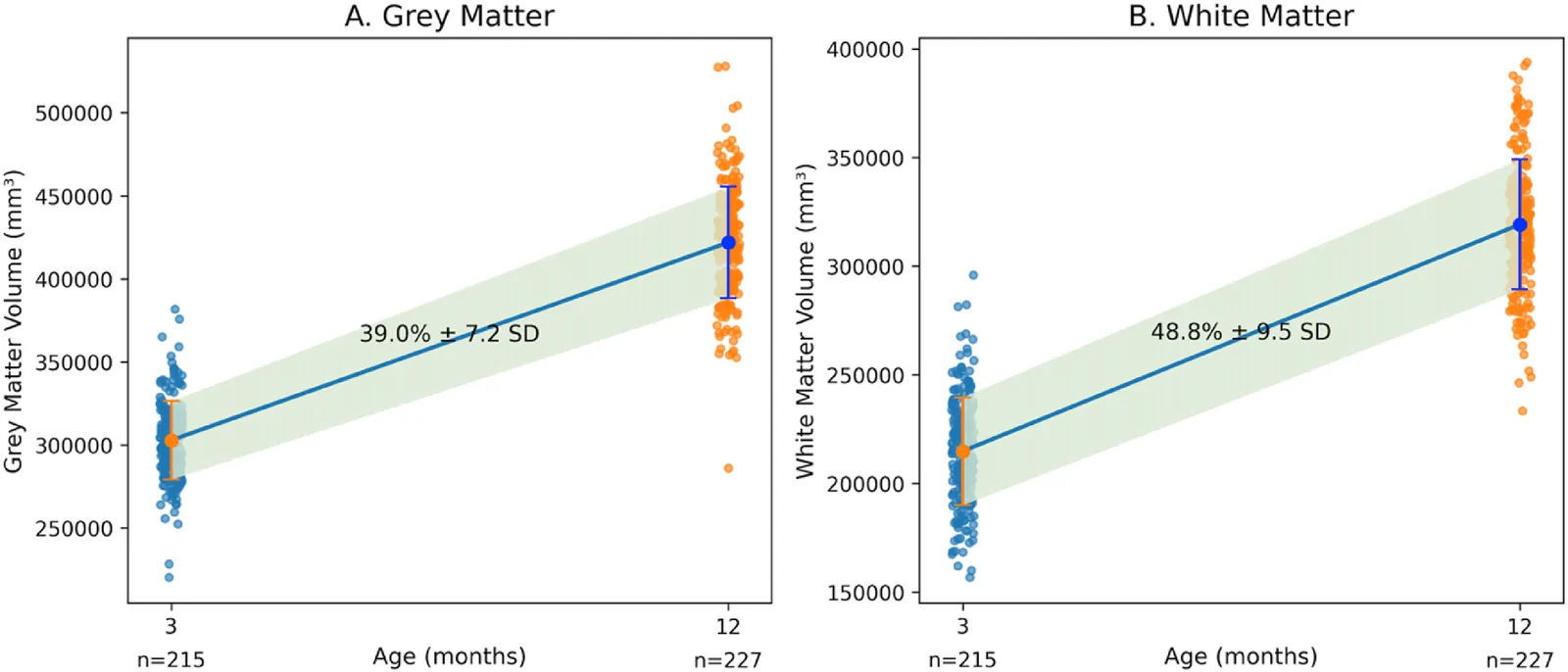
Grey matter (A) and white matter (B) volumes measured using ultra-low-field MRI at 3 and 12 months of age.

At 3 months of age, mean grey matter volume was 302,789 ± 23,599 mm³, increasing to 421,845 ± 33,629 mm³ at 12 months. Mean white matter volume increased from 214,762 ± 24,827 mm³ at 3 months to 319,168 ± 29,935 mm³ at 12 months (Table 3A). In paired longitudinal analyses (n = 99), grey matter volume increased from 300,388 ± 22,898 mm³ at 3 months to 417,491 ± 37,016 mm³ at 12 months, corresponding to a 39.0 ± 7.2% increase, while white matter volume increased from 214,846 ± 23,744 mm³ to 318,719 ± 32,395 mm³ (48.8 ± 9.5%) over the same interval (Fig. 4; Table 3B).

Participant-level grey and white matter volumes (mm³) derived from ultra-low-field MRI at 3 and 12 months of age. Individual points represent participant-level volumes. Solid lines indicate mean volumes at each timepoint, with shaded bands denoting ±1 standard deviation (SD). Sample sizes are shown beneath each timepoint (3 months: n = 215; 12 months: n = 227). Percentage changes represent mean within-participant longitudinal changes (± SD) calculated from infants with volumetric data available at both timepoints (n = 99).

Beyond grey and white matter, additional volumetric measures were also quantifiable, including the cerebellum, lateral ventricles, supratentorial tissue, and total intracranial volume (Table 3A). Paired longitudinal analyses demonstrated substantial age-related differences across all compartments, with cerebellar volume increasing by 83.9 ± 17.0%, supratentorial tissue by 43.0 ± 6.4%, and total intracranial volume by 45.0 ± 5.4% over the same interval (Table 3B). These observed differences between timepoints are consistent with expected patterns of early postnatal brain volume change reported in the literature [[36], [37], [38]], while primarily demonstrating that ULF-MRI datasets support quantification of biologically plausible variation across age.

**Fig. 5:**
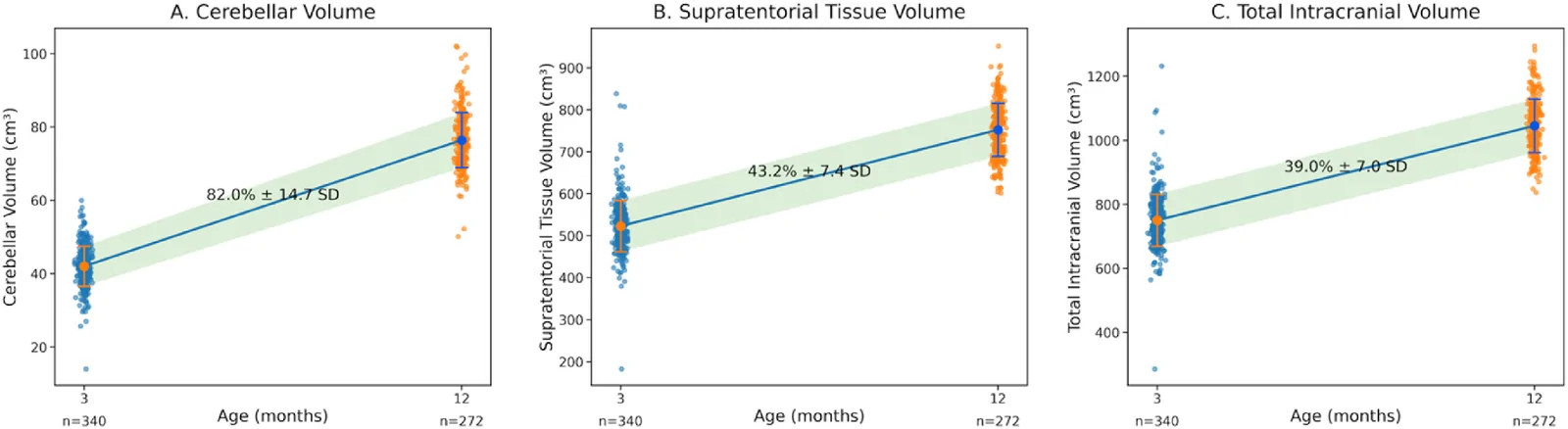
Cerebellar, supratentorial tissue, and total intracranial volumes derived from a parallel Flywheel-based volumetric analysis pipeline.

In addition to a separate volumetric processing workflow, a parallel cloud-based volumetric analysis pipeline implemented on the Flywheel platform was used to independently derive raw brain volume measures for a broader set of cortical, subcortical, and cerebrospinal fluid compartments. Using this alternative pipeline, cerebellar, supratentorial tissue, and total intracranial volumes demonstrated substantial age-related increases between 3 and 12 months (Fig. 5). Detailed cross-sectional summaries at each timepoint and paired longitudinal change estimates across multiple brain structures derived from the Flywheel pipeline are provided in Supplementary Table S5. Patterns of age-related change observed using the Flywheel-based analysis were consistent with those obtained from the separate processing workflow, supporting the analytic usability of ultra-low-field MRI data across independent processing environments, without constituting formal validation of specific processing pipelines.

Cerebellar volume (A), supratentorial tissue volume (B), and total intracranial volume (C) measured at 3 and 12 months of age using an independent, cloud-based Flywheel processing pipeline. Individual points represent participant-level raw volumetric measures displayed in cubic centimeters (cm³). Solid lines indicate mean volumes at each timepoint, with shaded bands and error bars denoting ±1 standard deviation (SD). Sample sizes are shown beneath each timepoint. Percent change changes represent mean within-participant longitudinal changes (± SD) calculated from infants with volumetric data available at both timepoints (n = 162). Source volumetric data were derived in mm³ and converted to cm³ for display only.

#### 3.3.2 Correspondence of volumetric estimates across processing workflows

As part of evaluating analytic usability, correspondence of volumetric estimates across independent processing workflows was assessed for cerebellar volume, supratentorial tissue volume, and total intracranial volume at 3 and 12 months of age using paired observations (Supplementary Table S6; Supplementary Fig. S2). At 3 months (n = 202 paired), strong positive correlations were observed for all three structures (cerebellum: r = 0.771, 95% CI 0.708–0.821; supratentorial tissue: r = 0.893, 95% CI 0.861–0.918; total intracranial volume: r = 0.916, 95% CI 0.890–0.936; all p < 0.001). Absolute agreement, assessed using two-way mixed-effects intraclass correlation coefficients (ICC[A,1]), was high for supratentorial tissue (ICC = 0.877, 95% CI 0.845–0.902) and moderate for total intracranial volume (ICC = 0.740, 95% CI 0.721–0.755), while cerebellar volume demonstrated moderate agreement (ICC = 0.543, 95% CI 0.494–0.582).

At 12 months (n = 194 paired), correspondence between pipelines remained strong, with high correlations for cerebellum (r = 0.776, 95% CI 0.713–0.826), supratentorial tissue (r = 0.969, 95% CI 0.959–0.977), and total intracranial volume (r = 0.972, 95% CI 0.963–0.979; all p < 0.001) (Supplementary Table S6; Supplementary Fig. S2). Agreement at 12 months was very high for supratentorial tissue (ICC = 0.955, 95% CI 0.944–0.963) and total intracranial volume (ICC = 0.961, 95% CI 0.952–0.967). In contrast, cerebellar volume showed lower absolute agreement despite maintained correlation (ICC = 0.411, 95% CI 0.379–0.435). This pattern – high correlation accompanied by reduced absolute agreement – suggests systematic or proportional differences between pipelines for cerebellar segmentation, potentially reflecting structure-specific sensitivity to segmentation boundary definitions or algorithmic scaling rather than random measurement error. Accordingly, these findings support correspondence between processing workflows but do not imply interchangeability of cerebellar volumetric estimates across pipelines.

### 3.4 Radiologist-reported interpretability and findings

Structural findings were evaluated among MRI examinations classified as analysable by the independent radiologist review (n = 396). Across analysable examinations, radiologist-reported structural findings were overwhelmingly within expected limits across routinely assessed domains (Table 5). Brain volume was rated as normal in 394/396 scans (99.5%), ventricles were normal in 391/396 (98.7%), the corpus callosum was reported as present in 391/396 (98.7%), and the cerebellum was normal in 394/396 (99.5%). Major intracranial pathology was uncommon: no cases of malformations of gross cortical development, haemorrhage, or mass lesions were identified. A small number of examinations demonstrated focal findings, including infarct (3/396, 0.8%) and extra-axial collections (2/396, 0.5%) (Table 5). Radiology follow-up was recommended for 7 scans (1.8% of 396 analysable scans; or 1.6% of 426 reviewed scans), with recommendations slightly more frequent at 12 months (5/206, 2.4%) than at 3 months (2/220, 0.9%) (Table 4). These recommendations reflected radiologist judgment based on structural appearances rather than image quality limitations. Together, these findings indicate that, once deemed interpretable, infant ultra-low-field MRI examinations supported clinically meaningful structural assessment, with a low rate of unexpected pathology and infrequent recommendations for radiology follow-up.

**Table 4:** Radiologist interpretability, motion artifact burden, and follow-up recommendations by visit. Radiologist-reviewed interpretability, motion artifact presence, and follow-up recommendations for ultra-low-field MRI sessions at 3 and 12 months of age. The denominator comprises radiologist-reviewed MRI sessions overall (n = 426) and by visit (3 months/visit 9: n = 220; 12 months/visit 12: n = 206). “MRI analysable” is based on the radiologist interpretability rating. Reasons for non-analysability are summarized among non-analysable sessions only. Motion artifact is reported overall and stratified by analysability status. Follow-up recommendations are reported as n (%) of radiologist-reviewed sessions.

Measure	Overall (n = 426)	3 months (n = 220)	12 months (n = 206)
**MRI analysable**	396 (93.0%)	200 (90.9%)	196 (95.1%)
**MRI not analysable**	30 (7.0%)	20 (9.1%)	10 (4.9%)
• Incomplete sequences / limited diagnostic series†	17 (56.7%)	12 (60.0%)	5 (50.0%)
• Motion degraded / non-diagnostic†	11 (36.7%)	7 (35.0%)	4 (40.0%)
• Other / unclear†	2 (6.7%)	1 (5.0%)	1 (10.0%)
**Motion artifact present (any)**	77 (18.1%)	60 (27.3%)	17 (8.3%)
• Among analysable scans	51 / 396 (12.9%)	41 / 200 (20.5%)	10 / 196 (5.1%)
• Among non-analysable scans	26 / 30 (86.7%)	19 / 20 (95.0%)	7 / 10 (70.0%)
**Radiology follow-up recommended**	7 (1.6%)	2 (0.9%)	5 (2.4%)

Notes:

Values are n (%), unless otherwise specified. Percentages for reasons for non-analysability are calculated using the number of non-analysable scans as the denominator (†). Motion artifact was recorded independently of analysability and may be present in scans deemed analysable

**Table 5:** Radiologist-reported structural findings among analysable ultra-low-field MRI sessions. Radiologist-reported structural findings across assessed brain domains among analysable ultra-low-field MRI sessions. The denominator comprises radiologist-rated analysable scans (n = 396). Findings are reported as n/N (%) for each structural domain, including brain volume, ventricles, corpus callosum, cerebellum, malformations of cortical development, haemorrhage, mass lesion, infarct, and extra-axial collections.

Structural domain	Finding	n / N (%)
**Brain volume**	Normal	394 / 396 (99.5%)
	Reduced volume	2 / 396 (0.5%)
**Ventricles**	Normal	391 / 396 (98.7%)
	Dilated / enlarged	3 / 396 (0.8%)
	Other / variant	2 / 396 (0.5%)
**Corpus callosum**	Present	391 / 396 (98.7%)
	Other / limited visualization	5 / 396 (1.3%)
**Cerebellum**	Normal	394 / 396 (99.5%)
	Abnormal / limited assessment	2 / 396 (0.5%)
**Malformation of cortical development**	Present	0 / 396 (0.0%)
**Haemorrhage**	Present	0 / 396 (0.0%)
**Mass lesion**	Present	0 / 396 (0.0%)
**Infarct**	Present	3 / 396 (0.8%)
**Extra-axial collection**	Present	2 / 396 (0.5%)

Notes:

Denominator is radiologist-rated analysable MRI sessions (n = 396). “Other / variant” and “limited visualization” categories reflect radiologist free-text descriptors (e.g., mild prominence, normal variants, or technical limitations). No cases of malformations of cortical development, haemorrhage, or mass lesions were identified

## 4 Discussion

This study indicates that mobile 64 mT ultra-low-field MRI (ULF-MRI) can be feasibly implemented for non-sedated infant brain imaging in a low-resource setting in sub-Saharan Africa (SSA), with high scan uptake, high interpretability of imaging, and support for quantitative volumetric analysis. By combining pragmatic workflow design, locally delivered scanning, and complementary artifact-based and radiologist-led quality assessment applied as parallel frameworks of data usability together with independent processing workflows, the study provides a scalable model for generating brain-based structural measures in contexts where conventional high-field MRI remains largely inaccessible. These findings extend the role of low-field MRI from proof-of-concept deployment toward supporting longitudinal imaging capability in early life (Fig. 1).

### 4.1 Feasibility of ULF-MRI for pediatric neuroimaging in SSA

A central finding of this work is that infant neuroimaging without sedation is operationally feasible in a facility-based research setting with limited infrastructure. This extends prior evidence from adult and emergency care studies conducted primarily in high-income settings, confirming that ultra-low-field MRI can be adapted for use in pediatric research in low- and middle-income contexts [4,39]. Unlike adult low-field MRI applications, infant neuroimaging poses additional challenges related to motion, dependence on natural sleep, and limited tolerance for prolonged acquisition, underscoring the importance of demonstrating feasibility in this age group [40]. Importantly, scanning was implemented by a locally trained multidisciplinary team and integrated into an ongoing longitudinal trial platform with high visit-level uptake and minimal attrition, supporting the practicality of repeated infant imaging in this environment.

The mobile design of the MRI system and its ability to operate using standard electrical outlets with backup power were important for successful deployment [27,41]. While power interruptions and environmental variability were encountered, adaptive operational strategies – including uninterruptible power supply support, workflow adjustments, and scheduling aligned with infant sleep cycles – enabled consistent scanning throughput. As implementation of ultra-low-field MRI expands in resource-constrained settings, additional approaches to improving power resilience, including integration with alternative energy solutions such as solar-supported battery systems, may further enhance operational continuity in environments with unreliable electricity supply. Caregivers were able to remain close to the scanner during acquisition, supporting infant settling and scan acceptability. Together, these findings indicate that feasibility depended not only on scanner performance, but also on context-responsive operational management within a real-world clinical research environment.

### 4.2 Image quality, artifacts, and processing compatibility

A substantial proportion of completed MRI sessions met full-brain quality criteria, demonstrating that ultra-low-field MRI can generate analysable structural brain images in infancy. Consistent with previous reports [42], the system produced clinically meaningful images despite lower spatial resolution and tissue contrast than conventional 1.5 T or 3 T systems – which may limit applications requiring fine sulcal–gyral delineation or detailed subcortical segmentation [43]. Nevertheless, it supported quantification of major brain tissue compartments and biologically plausible age-related differences consistent with established patterns of early brain development [[36], [37], [38]].

The artifact profile observed – dominated by motion, plane-specific intensity inhomogeneity (coronal), and linear signal irregularities in the sagittal plane – was consistent with expectations for low-field, non-sedated infant imaging (Fig. 3) [3,8,44]. Artifact-based and radiologist-based quality assessment demonstrated that not all artifacts had equivalent impact on data usability. Motion emerged as the principal determinant of non-analysability, whereas other artifacts were frequently present without preventing interpretability or volumetric processing. The underlying mechanisms of the observed sagittal linear signal irregularities and coronal intensity inhomogeneity remain uncertain. The present study was not designed to determine their origin, and they cannot be attributed specifically to the ultra-low-field regime based on the available data. Artifact mitigation strategies – including consistent infant positioning, careful coil placement, and standardized pre-scan procedures – contributed to maintaining image quality across variable operating conditions [29,39]. These findings highlight the importance of quality control approaches that evaluate the impact of artifacts on data usability rather than excluding scans solely because artifacts are present.

### 4.3 Analytic usability: volumetry across structures and pipelines

Beyond feasibility and image quality, this study indicates analytic usability of ultra-low-field MRI for multi-structure volumetric assessment in infancy (Fig. 4). Volumetric measures were successfully derived not only for grey and white matter, but also for the cerebellum, supratentorial tissue, lateral ventricles, and total intracranial volume, with within-participant comparisons demonstrating expected volumetric differences between the 3- and 12-month imaging timepoints across all compartments. These findings indicate that ULF-MRI data can support multi-structure quantitative analysis beyond total intracranial volume alone. The observed volumetric differences indicate that the imaging and processing workflows capture biologically plausible structural variation suitable for quantitative analysis. As normative infant ultra-low-field volumetric references are not yet available, these findings demonstrate analytic capability rather than reference standards for brain volume assessment.

An additional strength of this work is the use of an independent, cloud-based volumetric processing workflow implemented on the Flywheel platform (Fig. 5). Volumetric estimates derived from this parallel workflow showed patterns of age-related change consistent with those obtained from a separate processing pipeline, supporting analytic correspondence across processing environments. Correspondence analyses indicated strong correlations between pipelines for cerebellar, supratentorial tissue, and total intracranial volumes at both 3 and 12 months. Agreement metrics further indicated close alignment for supratentorial tissue and total intracranial volume at 12 months. In contrast, cerebellar volumes showed lower agreement despite maintained correlation, suggesting systematic or proportional differences between pipelines rather than random measurement variability. Such structure-specific differences likely reflect sensitivity to segmentation boundary definitions or algorithmic scaling, as reported for automated segmentation approaches [45]. Accordingly, the results demonstrate broad analytic consistency rather than interchangeability between processing workflows, particularly for cerebellar volumetry. These findings highlight the importance of maintaining a consistent processing workflow within studies and assessing cross-pipeline correspondence during method development.

### 4.4 Radiologist interpretability and clinical relevance

Radiologist-led review indicated that most infant ULF-MRI examinations were considered interpretable, with the majority of reviewed scans rated as analysable and only a small fraction deemed non-diagnostic. Motion was strongly associated with non-analysability, reinforcing its role as the principal limiting factor for clinical interpretation. Among analysable scans, major intracranial pathology was rare, and radiologist-reported structural assessments were overwhelmingly within expected limits. While this study was not designed to estimate population prevalence of pathology, the findings indicate that ultra-low-field MRI can support clinically meaningful structural assessment, consistent with previous reports [4]. Because paired high-field MRI examinations were not available, this study evaluated ULF-MRI as a stand-alone imaging modality rather than its equivalence to conventional high-field MRI. Consistent with its technical capabilities, ULF-MRI supported assessment of major structural abnormalities and other clinically important findings but was not intended for confident evaluation of subtle structural abnormalities or advanced neuroimaging applications. The low proportion of scans requiring follow-up further supports its practical use in this setting.

### 4.5 Positioning relative to prior work and broader context

These findings align with and extend prior feasibility studies of ultra-low-field MRI conducted in adult, emergency, and neurological care settings [4,42]. The present study adds novel insight by focusing on a pediatric cohort in a low-income sub-Saharan African setting – an area previously underrepresented in the neuroimaging literature [22]. Importantly, imaging was conducted within a controlled research hospital environment, which facilitated implementation but may not fully generalize to district-level clinics or community health settings. Nonetheless, the demonstrated ability to integrate ULF-MRI into a longitudinal pediatric study, supported by locally trained staff and adaptive operational strategies, represents an important intermediate step toward broader deployment. Furthermore, the observed correspondence across independent processing workflows supports robustness of volumetric estimation without implying interchangeability, highlighting the importance of maintaining consistent analytic approaches in longitudinal or multi-site analyses.

Compared with high-field pediatric neuroimaging studies, ULF-MRI does not provide the spatial resolution, tissue contrast, or advanced sequence capabilities of conventional systems [3,46]. However, the high scan completion rates, strong interpretability, and successful volumetric analysis observed here indicate that ULF-MRI can provide informative structural brain data where high-field systems are unavailable. In settings where the absence of neuroimaging infrastructure has historically precluded brain-based measurement, low-field MRI represents a meaningful expansion of research capability rather than a direct replacement for high-field imaging. Within the context of maternal and infant iron-deficiency interventions underpinning the parent trial, these data establish a foundation for future analyses examining relationships between early-life exposures and brain development.

### 4.6 Considerations for technique, capacity, and sustainability

The use of non-sedated scanning balanced safety, practicality, and cultural acceptability [2,47,48]. Although this approach increases susceptibility to motion artifacts, avoiding sedation is particularly important in low-resource contexts where pediatric anesthesiology support is limited. The observed scan completion and image quality outcomes indicate that infant neuroimaging without sedation is achievable when supported by trained staff, standardized procedures, and infant-adapted scheduling.

The collaborative training and mentorship model adopted in this study underscores the importance of capacity building for sustainable MRI implementation [49,50]. Local operation of the scanner, routine quality control, and basic troubleshooting were achieved, although ongoing implementation continues to benefit from remote technical support. These findings highlight both the progress made and the need for continued investment in training, governance, and analytic infrastructure to support equitable expansion of low-field neuroimaging in sub-Saharan Africa and other underserved regions [42].

### 4.7 Limitations and future directions

Several limitations warrant consideration. First, although feasibility outcomes were strong, imaging was conducted within a controlled research setting with established infrastructure, which may not fully reflect routine health service conditions. Second, motion remained a key source of image degradation and may limit applications requiring higher anatomical precision. In addition, the present study did not investigate the underlying mechanisms of the sagittal linear signal irregularities and coronal intensity inhomogeneity observed during image quality assessment. Future methodological studies should investigate the origin of these artifacts and evaluate acquisition, hardware, and processing strategies that may reduce their occurrence. Third, not all completed sessions underwent radiologist review, and volumetric analyses were restricted to scans meeting full-brain quality criteria; future work may explore approaches, including deep learning methods, capable of accommodating partially degraded data [51]. Where feasible, incorporating expert review of segmentation outputs is recommended as an additional layer of quality assurance to support assessment of segmentation appropriateness. Fourth, correspondence analyses suggest possible structure-specific differences between processing workflows, emphasizing the need for continued methodological refinement in low-field MRI, consistent with prior work demonstrating variability across segmentation approaches and processing frameworks [52,53]. In addition, paired high-field MRI examinations were not available for the same participants; therefore, direct comparison of radiologist assessments or diagnostic findings between ultra-low-field and conventional high-field MRI, including evaluation of its relative diagnostic limitations, was beyond the scope of this study. Future studies incorporating paired acquisitions, where feasible, would enable more rigorous validation of diagnostic correspondence. However, such studies remain challenging in many low-resource settings where ultra-low-field and conventional high-field MRI systems are rarely co-located. Recent methodological developments in low-field MRI have begun to address challenges, including approaches for motion-tolerant reconstruction and segmentation in lower-resolution datasets [54,55]. As ultra-low-field MRI technologies and associated image-processing methods continue to evolve, broader implementation should also consider appropriate governance, validation, and regulatory frameworks according to the intended use and local context. Fifth, procurement, maintenance, supporting equipment, software, and venue-related costs were not collected prospectively; consequently, the present study cannot provide a formal estimate of the resources required for implementation, and future deployment studies should incorporate systematic, context-specific costing (Tables 3A, 3B).

**Table 3A:** Cross-sectional volumetric brain measures at 3 and 12 months of age Cross-sectional raw regional and global brain volumes (mm³) derived from ultra-low-field MRI at each visit among all infants with usable volumetric data at that timepoint. Values are reported as mean ± standard deviation (SD).

Structure	3 months (n = 215) mean ± SD	12 months (n = 227) mean ± SD
Grey matter	302,789 ± 23,599	421,845 ± 33,629
White matter	214,762 ± 24,827	319,168 ± 29,935
Cerebellum	47,550 ± 6,763	87,001 ± 9,169
Lateral ventricles	8,987 ± 2,665	14,498 ± 4,640
Supratentorial tissue	517,552 ± 44,298	741,013 ± 59,629
Total intracranial volume	710,660 ± 61,759	1,029,425 ± 81,547

Notes:

All volumes are reported in cubic millimetres (mm³).

Cross-sectional summaries include all infants with QC-passing volumetric data at each visit.

Denominators differ between timepoints due to visit-specific scan completion and quality control.

No normalization, scaling, or derived ratio metrics were applied.

**Table 3B:** Paired longitudinal volumetric brain measures between 3 and 12 months of age Paired longitudinal raw regional and global brain volumes (mm³) derived from ultra-low-field MRI among infants with volumetric data available at both visits (n = 99). Values at each timepoint are reported as mean ± standard deviation (SD). Percent and absolute changes are calculated from within-infant paired observations and are reported as mean ± SD.

Structure	3 months (paired n = 99) mean ± SD	12 months (paired n = 99) mean ± SD	Percent change mean ± SD	Mean absolute change (mm³) mean ± SD
Grey matter	300,388 ± 22,898	417,491 ± 37,016	39.0% ± 7.2%	117,103 ± 22,715
White matter	214,846 ± 23,744	318,719 ± 32,395	48.8% ± 9.5%	103,872 ± 18,838
Cerebellum	48,170 ± 5,803	88,084 ± 9,439	83.9% ± 17.0%	39,914 ± 7,001
Lateral ventricles	9,044 ± 2,852	14,737 ± 5,389	63.4% ± 25.0%	5,692 ± 3,343
Supratentorial tissue	515,234 ± 43,442	736,209 ± 65,846	43.0% ± 6.4%	220,975 ± 35,780
Total intracranial volume	710,021 ± 58,558	1,029,103 ± 87,239	45.0% ± 5.4%	319,082 ± 45,315

Notes:

All volumes are reported in cubic millimetres (mm³).

Paired n represents infants with volumetric data available at both 3- and 12-month visits.

Percent and absolute changes are calculated from within-infant paired observations.

Values are presented as mean ± standard deviation (SD).

No normalization, scaling, or derived ratio metrics were applied.

## 5 Conclusions

This study indicates that mobile ultra-low-field MRI can be implemented for infant brain imaging in a low-resource sub-Saharan African setting, with high operational uptake, interpretable image quality, and support for quantitative volumetric analysis across multiple brain structures. By establishing an end-to-end imaging, quality control, and analytic framework supported by local implementation and cross-pipeline correspondence assessment, this work demonstrates that brain-based measures of early development can be feasibly acquired and analysed in settings where conventional MRI unavailable. These findings provide an important step toward more equitable measurement of early brain development and support broader integration of neuroimaging into developmental neuroscience research across underserved settings.

## Disclosure

EL is now an employee of Philips, but this work was completed while he was employed at Lund University. SCLD Is a senior program officer at the Gates Foundation but was not involved in study design, data acquisition, or image processing.

## Funding

This research was supported in part by the Science for Africa Foundation through the Developing Excellence in Leadership, Training and Science in Africa (DELTAS Africa) programme [DEL-22-002], with support from the Wellcome Trust, the UK Foreign, Commonwealth & Development Office, and the EDCTP2 programme supported by the European Union. Additional support was provided by the Training and Research Unit of Excellence (TRUE) INV-010612 (Malawi maternal anemia trial), Malawi and the Ultra-Low-Field Neuroimaging in the Young (UNITY, INV-090982) initiative funded by the Gates Foundation.

The first author (MV) is a doctoral researcher in the African Leadership for Measuring Brain Health in Children and Adolescents (ALMA) programme under DELTAS Africa, a recipient of the 2025 International Society for Magnetic Resonance in Medicine-Gates Knowledge Exchange Fellowship, and Neuroscience scholar in the Oxford Centre for Integrative Neuroimaging (OxCIN) Global Scholars Programme (2024/25). His doctoral training also received support from the Centre of Excellence for Nutrition (CEN), North-West University, South Africa, Potchefstroom, through the 2022 PhD Support Programme.

The funders had no role in study design; data collection, analysis, or interpretation; manuscript preparation; or the decision to submit the work for publication. For the purpose of open access, the author has applied a CC BY public copyright license to any Author Accepted Manuscript version arising from this submission.

## CRediT authorship contribution statement

**Maclean Vokhiwa:** Writing – review & editing, Writing – original draft, Visualization, Project administration, Methodology, Investigation, Formal analysis, Data curation, Conceptualization. **Karen Chetcuti:** Writing – review & editing, Investigation. **Frederik Lange:** Writing – review & editing, Supervision, Investigation. **Niall J. Bourke:** Writing – review & editing, Investigation. **Louise Randall:** Investigation. **Steven Greenstein:** Investigation. **Marc Seal:** Writing – review & editing, Investigation. **Richard Beare:** Investigation. **Adam Hussain:** Investigation. **Padma Rao:** Investigation. **Emil Ljungberg:** Writing – review & editing, Investigation. **Francesco Padormo:** Writing – review & editing, Investigation. **John Rogers:** Investigation. **Pip Torelli:** Investigation. **Steve Williams:** Investigation, Funding acquisition. **Derek K. Jones:** Writing – review & editing, Funding acquisition. **Sean C.L. Deoni:** Writing – review & editing. **Sant-Rayn Pasricha:** Funding acquisition. **Kamija S. Phiri:** Writing – review & editing, Supervision, Funding acquisition. **Eric Umar:** Writing – review & editing, Supervision.

## Declaration of competing interest

The authors declare the following financial interests/personal relationships which may be considered as potential competing interests Emil Ljungberg reports a relationship with Phillips that includes: employment. Sean C. L. Deoni reports a relationship with The Gates Foundation that includes: employment. If there are other authors, they declare that they have no known competing financial interests or personal relationships that could have appeared to influence the work reported in this paper.

## Data Availability

Data will be made available on request.

## Appendix Supplementary materials

Image, application 1
