# Supplementary material for: Feasibility, image quality, and analytic usability of mobile 64 mT ultra-low-field MRI for infant brain imaging at 3 and 12 months in southern Malawi
Source: J Magn Reson Open. 2026 Sep;28:None. doi: 10.1016/j.jmro.2026.100225 (PMC13569786; doi:10.1016/j.jmro.2026.100225)

Supplementary Materials

Title:
Feasibility, Image Quality, and Analytic Usability of Mobile 64 mT Ultra-Low-Field MRI for Infant Brain Imaging at 3 and 12 Months in Southern Malawi

DOI: 10.1016/j.jmro.2026.100225

Running Title: Ultra-Low-Field MRI for Infant Brain Imaging in Malawi

Authors:
Maclean Vokhiwa[1 2 *], Karen Chetcuti[3], Frederik Lange[4], Niall J. Bourke[5], Louise Randall[6], Steven Greenstein[7], Marc Seal[7], Richard Beare[7], Adam Hussain[8], Padma Rao[8], Emil Ljungberg[9 10], Francesco Padormo[11], John Rogers[11], Pip Torelli[11], Steve Williams[5], Derek K. Jones[12], Sean C. L. Deoni[13], Sant-Rayn Pasricha[6], Kamija S. Phiri[1 2], Eric Umar[2], UNITY consortium[14]

Affiliations
[1] Training & Research Unit of Excellence (TRUE), Blantyre, Malawi
[2] Kamuzu University of Health Sciences (KUHeS), Blantyre, Malawi
[3] Department of Radiology, Kamuzu University of Health Sciences (KUHeS), Blantyre, Malawi
[4] Centre for Integrative Neuroimaging (OxCIN), FMRIB, Nuffield Department of Clinical Neurosciences, University of Oxford, United Kingdom
[5] Centre for Neuroimaging Sciences, Department of Psychology and Neuroscience, King's College London, London, United Kingdom
[6] Population Health and Immunity, Walter and Eliza Hall Institute of Medical Research, Parkville, Victoria, Australia
[7] Developmental Imaging, Murdoch Children's Research Institute, The Royal Children's Hospital, Victoria, Australia
[8] Department of Medical Imaging, The Royal Children's Hospital, Parkville, Victoria, Australia
[9] Department of Neuroimaging, King's College London, London, United Kingdom
[10] Department of Medical Radiation Physics, Lund University, Lund, Sweden
[11] Hyperfine, Inc., Guilford, CT, USA
[12] Cardiff University Brain Research Imaging Centre (CUBRIC), Cardiff University, Cardiff, United Kingdom
[13] Maternal, Newborn, Child Nutrition & Health, Gates Foundation, Seattle, WA, United States
[14] Ultra-Low-Field Neuroimaging in The Young (UNITY), United Kingdom

Corresponding Author
Maclean Vokhiwa, MPH
Kamuzu University of Health Sciences Private Bag 360, Chichiri, Blantyre 3, Malawi
Email: mvokhiwa@gmail.com
Phone: +265999080096

Table of Contents

1. Supplementary Table S1 — MRI acquisition parameters......3

2. Supplementary Table S2 — Infant MRI sessions and operational characteristics (A - C)......4

3. Supplementary Table S3 — Operational implementation challenges......5

4. Supplementary Figure S1. Dust and debris accumulation within the scanner integrated computer module identified following a temporary operational interruption. 6

5. Supplementary Table S4 — Data pipeline disruptions......7

6. Supplementary Table S5 — Flywheel-derived volumetric brain measures......8

7. Supplementary Table S6 — Correspondence between processing pipelines......9

8. Supplementary Figure S2 — Correspondence scatterplots......10

1. Supplementary Table S1 — MRI acquisition parameters

Sequence-specific acquisition parameters, acquisition times, and intended purpose for each sequence included in the standardized 64 mT ultra-low-field MRI protocol used for non-sedated infant brain imaging. The protocol was designed to provide complete orthogonal structural brain coverage while maintaining a short acquisition time suitable for non-sedated infant imaging, thereby supporting image quality assessment, radiologist review, and volumetric analyses.

Sequence	TR/TE (ms)	Voxel size (mm³)	Matrix size	Time (min)	Purpose
Localizer	8.71 / 6.53	3.5 × 3.55 × 3.5	62 x 62 x 62	0.35	Scan planning and positioning
T2 Axial (Fast)	2000.00/182.4	1.6 x 1.6 x 5.0	112 x 136 x 36	2.09	Primary structural imaging; image quality assessment; volumetric processing
T2 Coronal (Fast)	2000.00/217.6	1.6 x 5.0 x 1.6	112 x 44 x 112	2.07	Orthogonal structural coverage; image quality assessment; volumetric processing
T2 Sagittal (Fast)	2000.00/230.00	5.0 x 1.6 x 1.6	36 x 136 x 112	1.50	Orthogonal structural coverage; image quality assessment; volumetric processing
T1 axial Standard	880.0/5.96	1.6 × 1.6 × 5.0	112 x 138 x 36	4.01	Complementary anatomical information for structural assessment and radiologist review

Abbreviations: TR/TE = repetition time/echo time, values are reported in milliseconds

2. Supplementary Table S2 — Infant MRI sessions and operational characteristics (A - C)

Descriptive and operational summary of infant MRI sessions by visit, including:

A. Participant and visit characteristics
B. Infant behavioral state during completed MRI sessions
C. Sequence-specific acquisition success among completed MRI sessions

This table provides detailed operational context to complement the feasibility outcomes reported in the main manuscript.

Percentages were calculated using visit-specific denominators unless otherwise stated. Infant behavioral state was recorded by the scanning team at the conclusion of each completed MRI session. Sequence completion reflects successful acquisition during completed scan sessions and does not account for post-acquisition image quality assessment, which is reported separately in the main Results.

**A. Participant and visit characteristics**		
Characteristic	3 months	12 months
Eligible visits, n	410	400
MRI completed, n (%)	355 (86.6)	299 (74.8)
Female, n (%) *	169 (49.9; n = 339)	132 (50.0; n = 264)
Age at visit, days (mean ± SD) **	91 ± 6	365 ± 7
Age range, days**	84–103	354–378
**B. Infant behavioral state during completed MRI sessions**		
	3 months	12 months
Sleeping throughout	279 (78.6)	211 (70.6)
Intermittent movement	60 (16.9)	63 (21.1)
Crying / unsettled	16 (4.5)	25 (8.4)
Total completed scans	355	299
**C. Sequence-specific acquisition success among completed MRI sessions**		
Sequence	3 months, % completed	12 months, % completed
Localizer	94.6	95.0
Axial T2-weighted	99.4	99.7
Sagittal T2-weighted	95.8	96.7
Coronal T2-weighted	96.6	97.0
Axial T1-weighted	91.0	95.0

* Percentages were calculated among participants with available sex information. Values shown as "n =" indicate the denominator used for each percentage at the corresponding timepoint.
**Calculated among records with available age data.

3. Supplementary Table S3 — Operational implementation challenges

Summary of operational challenges and mitigation strategies identified during weekly implementation review meetings across the infant ultra-low-field MRI study period. Observations were categorized thematically to summarize practical implementation experiences and adaptive responses relevant to study feasibility, acceptability, and workflow sustainability. This table complements the feasibility outcomes reported in the main Results.

Implementation domain	Representative observation(s)	Operational impact	Mitigation implemented	Resolution status
Participant flow & scheduling	Higher-than-anticipated attendance on specific clinic days; late arrival of participants from distant catchment areas	Increased waiting times; reduced likelihood of infant sleep prior to scanning	Adjustment of daily booking targets; prioritization of imaging earlier in clinic flow; caregiver counseling on arrival timing	Ongoing
Infant settling & comfort	Infants unsettled due to prolonged waiting or environmental stimulation	Scan delays or non-completion	Noise minimization, dimmed lighting, swaddling, caregiver presence, provision of comfort items	Ongoing
Power supply & infrastructure	Intermittent mains power; reliance on temporary power sources during early setup	Risk of scan interruption; delayed sessions	Installation and use of UPS; coordination with generator backup	Resolved
Scan room environment	Elevated room temperatures during warmer periods	Infant discomfort; difficulty settling	Temporary cooling solutions (mobile air conditioning); schedule adjustments	Resolved
Data transfer & image management	Incomplete image transfer to PACS/cloud; synchronization delays	Delayed quality checks and downstream processing	Workflow adjustments; reconnection procedures; coordination with technical support	Resolved
Consent & caregiver acceptability	Concern that caregivers perceived imaging as mandatory; early departures before assessments	Risk to voluntary participation and retention	Reinforcement of consent language; staff retraining on optional participation; workflow streamlining	Ongoing
Staffing & workflow coordination	Bottlenecks during concurrent assessments and imaging	Reduced throughput on high-volume days	Task redistribution; clearer role allocation during clinic flow	Ongoing
Equipment & workspace setup	Limited furniture or workspace in early setup phase	Inefficient workflow; temporary setup constraints	Procurement and interim workspace solutions	Resolved

4. Supplementary Figure S1. Dust and debris accumulation within the scanner integrated computer module identified following a temporary operational interruption.

Photographs documenting accumulation of dust and environmental debris within the power supply unit of the scanner's integrated computer module, identified during inspection following a temporary operational interruption. Inspection suggested that dust ingress was associated with local environmental conditions and airflow from adjacent windows. The issue was resolved through cleaning of affected components, improved environmental sealing, and replacement of the manufacturer-supplied power supply unit. Scanner performance was fully restored, and no further interruptions occurred during the remainder of the study period.

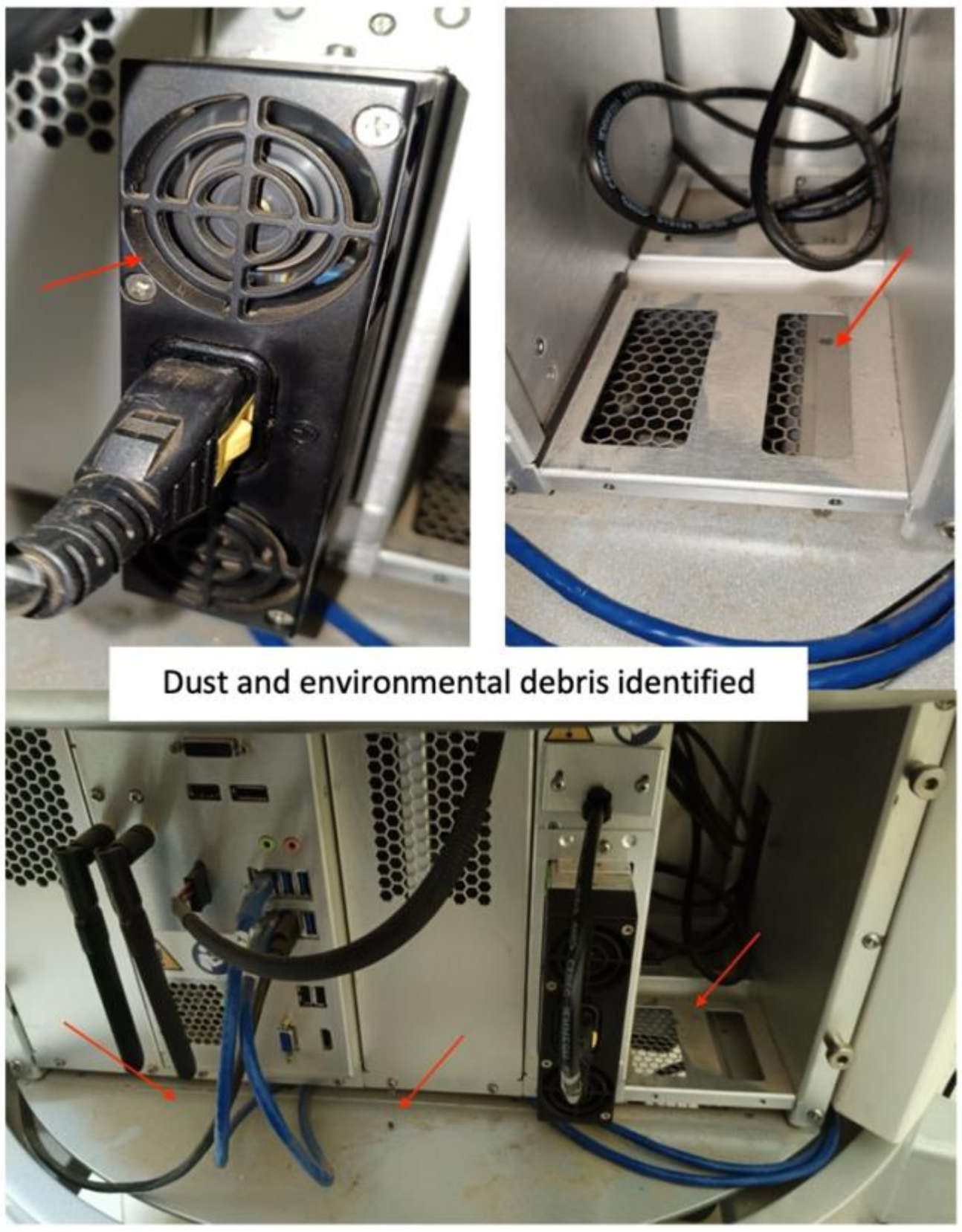

5. Supplementary Table S4 — Data pipeline disruptions

Summary of data pipeline disruptions and mitigation strategies documented during weekly operational review meetings across the infant ultra-low-field MRI study period. The table summarizes challenges and corresponding resolutions across the imaging data lifecycle, including image acquisition, data transfer, metadata entry, and data reconciliation. This table complements the feasibility outcomes reported in the main Results.

Pipeline stage	Nature of disruption	Operational impact	Mitigation implemented	Resolution status
Image acquisition → local storage	Completed scans not consistently appearing in local storage immediately after acquisition	Delayed confirmation of scan completeness	Post-scan verification checklist introduced; reconciliation against daily scan logs	Resolved
Local storage → PACS / scanner server	Intermittent failure of automatic transfer of completed scan series	Delayed quality review and downstream processing	Manual reconciliation of missing series; reconnection to scanner server; coordination with technical support	Resolved
PACS / scanner server → cloud platform	Incomplete or delayed synchronization of image data to cloud-based platform	Temporary gaps in centralized image archive	Workflow adjustments; scheduled synchronization checks; technical troubleshooting	Resolved
Imaging data ↔ CRF metadata linkage	Mismatch between imaging files and CRF entries due to delayed or incomplete data entry	Risk of misalignment between imaging and participant metadata	Reinforcement of CRF completion procedures; implementation of cross-checks between scan logs and CRF records	Resolved
CRF (REDCap) data entry	Delayed completion or correction of CRF fields during high-volume clinic days	Temporary data incompleteness	Staff retraining; task redistribution; use of completion checklists	Ongoing early, later resolved
End-to-end data reconciliation	Inconsistencies between scan logs, stored images, and CRF records identified during review	Potential omission of scan sessions from analytic datasets	Weekly reconciliation procedures instituted as part of operational review meetings	Resolved

6. Supplementary Table S5 — Flywheel-derived volumetric brain measures

Flywheel-derived volumetric brain measures (mm³) across cortical, subcortical, and cerebrospinal fluid compartments at 3 and 12 months of age. Values at each timepoint are reported as mean ± standard deviation (SD) among infants with volumetric data available at both visits (n = 162). Percent change and mean absolute change were calculated from within-participant observations and are reported as mean ± SD. All volumes are reported in mm³ without normalization or derived ratio metrics. These data summarize volumetric outputs generated by the Flywheel-based processing pipeline and complement the correspondence analyses presented in the main manuscript.

Structure	3 months (paired n = 162) mean ± SD	12 months (paired n = 162) mean ± SD	Percent change mean ± SD	Mean absolute change (mm³) mean ± SD
Anterior callosum	5,115 ± 606	5,682 ± 834	11.7% ± 15.3%	567 ± 764
Brainstem	7,495 ± 887	11,403 ± 1,672	52.5% ± 15.8%	3,908 ± 1,247
Brainstem CSF	6,538 ± 929	10,121 ± 1,733	55.4% ± 19.6%	3,583 ± 1,305
Central callosum	1,158 ± 162	1,302 ± 259	14.0% ± 24.1%	144 ± 270
Cerebellum	42,355 ± 5,074	76,733 ± 8,064	82.0% ± 14.7%	34,379 ± 5,389
Cerebellum CSF	15,819 ± 1,858	22,160 ± 2,140	41.2% ± 15.1%	6,341 ± 2,324
Left caudate	2,003 ± 287	2,347 ± 263	18.5% ± 15.0%	344 ± 347
Left globus pallidus	1,018 ± 113	1,242 ± 116	22.8% ± 12.2%	224 ± 104
Left putamen	2,165 ± 230	3,375 ± 350	56.5% ± 13.0%	1,210 ± 251
Left thalamus	5,402 ± 486	5,986 ± 517	11.2% ± 9.2%	584 ± 479
Mid-anterior callosum	2,309 ± 310	2,061 ± 349	−9.5% ± 17.2%	−247 ± 418
Mid-posterior callosum	854 ± 117	975 ± 184	15.2% ± 21.3%	121 ± 176
Posterior callosum	4,773 ± 710	6,017 ± 916	27.1% ± 17.7%	1,245 ± 788
Right caudate	2,021 ± 278	2,391 ± 256	19.6% ± 14.8%	370 ± 313
Right globus pallidus	992 ± 110	1,259 ± 110	27.9% ± 13.0%	267 ± 109
Right putamen	2,484 ± 270	3,604 ± 364	45.9% ± 14.0%	1,119 ± 306
Right thalamus	5,324 ± 486	5,838 ± 457	10.1% ± 8.8%	514 ± 455
Ventricles	12,687 ± 3,197	17,242 ± 6,735	35.7% ± 33.0%	4,555 ± 4,996
Supratentorial CSF	156,164 ± 15,123	174,535 ± 18,690	12.0% ± 9.3%	18,371 ± 14,205
Supratentorial tissue	526,572 ± 46,364	752,957 ± 66,152	43.2% ± 7.4%	226,384 ± 38,726
Total intracranial volume	754,943 ± 63,181	1,047,909 ± 90,095	39.0% ± 7.0%	292,966 ± 53,247

Notes:
All volumes are reported in cubic millimetres (mm³).
Paired n represents infants with volumetric data available at both 3- and 12-month visits.
Percent and absolute changes are calculated from within-infant paired observations.
Values are presented as mean ± standard deviation (SD).
No normalization, scaling, or derived ratio metrics were applied.

7. Supplementary Table S6 — Correspondence between processing pipelines

Correspondence between volumetric brain measures derived from a separate processing pipeline and a Flywheel-based processing pipeline for cerebellar volume, supratentorial tissue volume, and total intracranial volume at 3 and 12 months of age. Metrics include Pearson correlation coefficients (linear association) and two-way mixed-effects intraclass correlation coefficients (ICC[A,1]; absolute agreement). Analyses are based on matched volumetric estimates derived from the same MRI examinations processed independently through the two processing pipelines. All volumes are reported in mm³.

Structure	Timepoint	Paired n	Pearson r (95% CI)	p-value	ICC(A,1) (95% CI)
Cerebellum	3 months	202	0.771 (0.708–0.821)	<0.001	0.543 (0.494–0.582)
Supratentorial tissue	3 months	202	0.893 (0.861–0.918)	<0.001	0.877 (0.845–0.902)
Total intracranial volume	3 months	202	0.916 (0.890–0.936)	<0.001	0.740 (0.721–0.755)
Cerebellum	12 months	194	0.776 (0.713–0.826)	<0.001	0.411 (0.379–0.435)
Supratentorial tissue	12 months	194	0.969 (0.959–0.977)	<0.001	0.955 (0.944–0.963)
Total intracranial volume	12 months	194	0.972 (0.963–0.979)	<0.001	0.961 (0.952–0.967)

Note: Matched observations represent volumetric estimates derived from the same MRI examination processed independently through the separate processing workflow and Flywheel-based processing workflow.

8. Supplementary Figure S2 — Correspondence scatterplots

Scatter plots illustrating correspondence between volumetric estimates derived from a separate processing pipeline and a Flywheel-based processing pipeline for cerebellar volume, supratentorial tissue volume, and total intracranial volume at 3 months (top row) and 12 months (bottom row). Each point represents matched volumetric estimates derived from the same MRI examination processed independently through the two processing pipelines, with identity lines indicating perfect agreement. Pearson correlation coefficients and intraclass correlation coefficients corresponding to these relationships are reported in Supplementary Table S6. Volumes are displayed in cubic centimetres (cm³) for visual clarity; source volumetric data were derived in mm³ and converted to cm³ for display only.

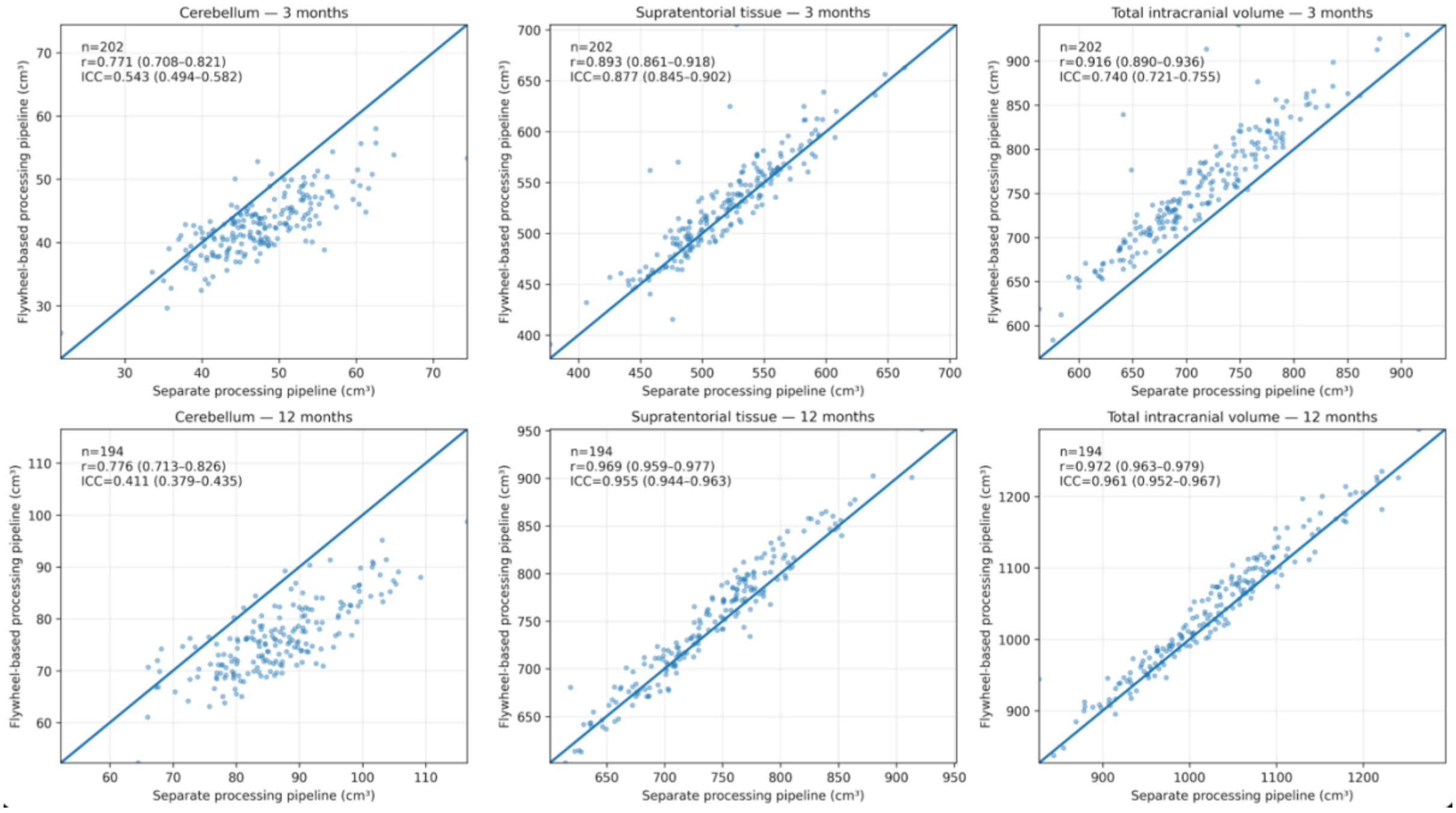

Supplement: Supplementary file 1 [file mmc1.pdf]
